# Transformation of non-neuritic into neuritic plaques during AD progression drives cortical spread of tau pathology via regenerative failure

**DOI:** 10.1186/s40478-023-01688-6

**Published:** 2023-12-01

**Authors:** Wangchen Tsering, Gabriela P. Hery, Jennifer L. Phillips, Kiara Lolo, Tim Bathe, Jonathan A. Villareal, Isabelle Y. Ruan, Stefan Prokop

**Affiliations:** 1 Center for Translational Research in Neurodegenerative Disease, College of Medicine, University of Florida, Gainesville, FL USA; 2 Department of Neuroscience, College of Medicine, University of Florida, Gainesville, FL USA; 3 Department of Pathology, College of Medicine, University of Florida, Gainesville, FL USA; 4 College of Medicine, Mcknight Brain Institute, University of Florida, Gainesville, FL USA; 5 Fixel Institute for Neurological Diseases, University of Florida, Gainesville, FL USA

**Keywords:** Neuritic plaque, Alzheimer’s disease, Spatial transcriptomics, Dystrophic neurites, Regenerative failure

## Abstract

Extracellular amyloid-β (Aβ) plaques and intracellular aggregates of tau protein in form of neurofibrillary tangles (NFT) are pathological hallmarks of Alzheimer’s disease (AD). The exact mechanism how these two protein aggregates interact in AD is still a matter of debate. Neuritic plaques (NP), a subset of Aβ plaques containing dystrophic neurites (DN), are suggested to be unique to AD and might play a role in the interaction of Aβ and tau. Quantifying NP and non-NP in postmortem brain specimens from patients with increasing severity of AD neuropathological changes (ADNC), we demonstrate that the total number of Aβ plaques and NP increase, while the number of non-NP stagnates. Furthermore, investigating the correlation between NP and NFT, we identified unexpected brain region-specific differences when comparing cases with increasingly more severe ADNC. In neocortical regions NFT counts increase in parallel with NP counts during the progression of ADNC, while this correlation is not observed in hippocampus. These data support the notion that non-NP are transformed into NP during the progression of ADNC and indicate that NP might drive cortical NFT formation. Next, using spatial transcriptomics, we analyzed the gene expression profile of the microenvironment around non-NP and NP. We identified an upregulation of neuronal systems and Ca-dependent event pathways around NP compared to non-NP. We speculate that the upregulation of these transcripts may hint at a compensatory mechanism underlying NP formation. Our studies suggest that the transformation of non-NP to NP is a key event in ADNC progression and points to regenerative failure as a potential driving force of this process.

**Supplementary Information:**

The online version contains supplementary material available at 10.1186/s40478-023-01688-6.

## Introduction

Alzheimer’s disease (AD) is neuropathologically characterized by the presence of extracellular amyloid- β deposits (Aβ), intracellular neurofibrillary tangles (NFT), and neuronal loss [70]. Genetic studies indicate that Aβ is central to AD pathophysiology [62, 69]. According to the Amyloid Cascade Hypothesis (ACH), Aβ plaques are the main pathological trigger of tau pathology, leading to neurodegeneration and cognitive decline [62]. The ACH, however, does not account for different morphologies of Aβ plaques observed in human AD brains and does not consider how subtypes of Aβ plaques may differentially affect the cascade of events leading to tauopathy and neurodegeneration. A plethora of Aβ plaque morphologies have been described in the scientific literature, including but not limited to “neuritic plaques,” “dense-core plaques,” “diffuse plaques,” “burned-out plaques,” “cotton-wool plaques,” [16] “coarse-grain plaques” [8], and “bird nest plaques” [32]. Furthermore, Aβ deposits are also detected in cognitively normal, elderly subjects, [19] a condition termed “pathological aging”. The majority of animal models of Aβ deposition however fail to recapitulate the diversity of the Aβ plaque morphologies observed in human brain tissue, which may explain the limited knowledge about the contributions of individual Aβ plaque subtypes to AD pathophysiology.

Neuritic plaques (NP), a subset of Aβ plaques surrounded by swollen or dystrophic neurites (DN), are considered to be unique to AD. Abnormal DN are heterogeneous and may contain inclusions such as p-tau, mitochondria, paired helical filaments, axonal transport proteins, and multivesicular bodies [12, 15, 23, 27, 64]. Swollen neurites are thought to be mostly axonal cytoskeleton alterations [9, 43, 56, 67] and have been demonstrated to be associated with dysregulated calcium homeostasis [36, 68]. DN can be found around both dense-cored plaques and diffuse plaques, with 80% of DN being associated with dense-core plaques in end-stage AD cases, as compared to the 20% of DN being associated with diffuse plaques [21]. The mechanism underlying DN formation and its physiological consequences is not yet known. A study using the 5xFAD mouse model of Aβ pathology injected with human brain-derived pathological tau (AD-tau) suggested that DN are the site where Aβ plaques facilitate tau accumulation [30]. However, evidence of this process in human AD brains is lacking.

NP are sites where important neuropathological features of AD converge – namely, Aβ plaques, tau aggregates, and activated glial cells. Moreover, NP are shown to disrupt long-range neuronal networks [79] and are positively correlated with cognitive decline [29, 45]. Therefore, understanding the distribution of NP and non-NP in different brain regions during the progression of AD neuropathological changes is imperative.

Here, we used postmortem human brain tissues from cases with progressively worse Alzheimer’s disease neuropathological changes (ADNC) to examine the relationship between non-NP, NP, and NFT. We next employed spatial transcriptomics to evaluate the gene expression profile of microenvironment around non-NP and NP. Our results suggest that non-NP are transformed into NP during the progression of ADNC and indicate that NP are responsible for the cortical spread of NFT via regenerative failure of degenerating neurons.

## Methods

### Patient samples

For this study, 83 postmortem brain tissues were selected from the University of Florida Human Brain and Tissue Bank (UF HBTB) (Table 1). All protocols were approved by the University of Florida Institutional Review Board. The NIA-AA guideline for pathological diagnosis constitutes the Thal phase of Aβ plaques (A), Braak and Braak NFT stage (B), and CERAD neuritic plaque score (C) – “ABC” score – for determining ADNC [50]. Based on the NIA-AA guideline for the neuropathological assessment of AD [31], cases were grouped into “low AD” (18 cases), “interm AD” (22 cases), and “high AD” (43 cases). High AD cases were further separated into cases with “pure” Aβ and tau pathology (21 cases) and cases with additional Lewy body pathology (“high AD mixed pathology,” 22 cases). Hippocampus, adjacent portions of inferior temporal cortex, frontal cortex, and occipital cortex were analyzed for each case. The postmortem brain tissue samples were balanced for sex. Details of case demographics and neuropathological data are shown in Table 1 and Additional File 1: Table 2.

**Table 1:** Neuropathological data for cases used in this study

Sample	Neuropath	Thal	Braak	CERAD	APOE	Sex	Age	PMI	MMSE score	Lewy body pathology	LATE-NC
1	Low AD	1	I	None	3/3	F	72	144	NA	No	No
2	Low AD	1	I	None	3/3	F	65	15	NA	No	No
3	Low AD	1	III	None	3/4	F	79	72	NA	No	No
4	Low AD	1	III	None	3/3	F	92	8	NA	No	No
5	Low AD	1	IV	None	2/3	F	74	7.5	NA	No	No
6	Low AD	2	II	None	3/4	F	63	168	NA	No	No
7	Low AD	2	III	None	3/4	F	82	5	NA	No	No
8	Low AD	3	II	Sparse	3/3	F	96	16	NA	No	No
9	Low AD	4	II	Sparse	2/3	F	77	12	30/30	No	No
10	Low AD	4	0	None	3/4	F	82	22	20/30	No	No
11	Low AD	4	I	None	3/3	F	66	72	NA	No	No
12	Low AD	1	I	None	2/3	M	68	48	NA	No	No
13	Low AD	1	II	None	3/3	M	89	28	NA	No	No
14	Low AD	3	II	mild	3/3	M	91	72	NA	No	No
15	Low AD	3	II	Sparse	3/3	M	81	144	NA	No	No
16	Low AD	1	II	None	3/3	M	90	14	NA	No	No
17	Low AD	1	II	None	3/3	M	88	4	19/30	No	No
18	Low AD	3	II	Sparse	3/3	M	79	34	unspecific dementia	No	No
19	Intermediate AD	4	III	Moderate	3/3	F	83	192	NA	No	No
20	Intermediate AD	5	IV	Frequent	3/3	F	100	18	NA	No	No
22	Intermediate AD	3	II	Sparse	3/3	F	79	?	NA	No	No
23	Intermediate AD	5	IV	Frequent	3/3	F	93	18	NA	No	stage 2
24	Intermediate AD	4	IV	Frequent	3/3	F	89	8	24/30	No	No
25	Intermediate AD	4	III	moderate	3/3	F	106	4	NA	No	No
26	Intermediate AD	3	V	Frequent	3/3	F	83	27	NA	No	No
27	Intermediate AD	3	IV	moderate	3/4	F	74	192	NA	No	No
28	Intermediate AD	4	V	moderate	3/4	F	99	8	NA	No	No
29	Intermediate AD	4	IV	Frequent	3/3	F	90	19	MOCA- 22/30	No	No
30	Intermediate AD	5	IV	Frequent	3/3	M	72	72	NA	No	No
31	Intermediate AD	5	IV	moderate	3/3	M	86	48	NA	No	No
33	Intermediate AD	5	III	sparse	2/4	M	78	4	MOCA- 11/29	No	No
34	Intermediate AD	4	III	sparse	3/4	M	86	12	NA	No	No
35	Intermediate AD	4	III	moderate	3/3	M	72	17	30/30	No	No
36	Intermediate AD	5	IV	Frequent	3/4	M	90	7	NA	No	No
37	Intermediate AD	3	VI	moderate	3/3	M	82	13	1/30	No	No
38	Intermediate AD	5	III	moderate	3/3	M	83	22	NA	No	No
39	Intermediate AD	5	IV	moderate	3/3	M	78	10	NA	No	stage 2
40	Intermediate AD	3	IV	sparse	3/3	M	73	8	NA	No	stage 2
21	High AD Pure	5	VI	moderate	2/3	F	68	9	NA	No	No
32	High AD Pure	5	VI	moderate	3/3	M	73	7	NA	No	No
41	High AD Pure	5	V	Frequent	2/4	F	97	10	NA	No	No
42	High AD Pure	5	VI	Frequent	3/3	F	63	10	NA	No	No
43	High AD Pure	5	V	Frequent	3/3	F	83	9	NA	No	No
44	High AD Pure	5	V	Frequent	3/3	F	78	5	NA	No	No
45	High AD Pure	5	V	Frequent	3/3	F	64	12	NA	No	No
46	High AD Pure	5	VI	Frequent	3/3	F	76	3.5	dementia reported	No	No
47	High AD Pure	5	VI	Frequent	3/3	F	80	19	NA	No	No
48	High AD Pure	4	V	Frequent	3/4	F	85	18	23/30	No	No
49	High AD Pure	5	V	Frequent	3/4	F	72	12	NA	No	No
50	High AD Pure	5	V	Frequent	3/4	F	86	14	13/30	No	No
62	High AD Pure	4	V	Frequent	3/4	M	84	16	9/30	No	No
63	High AD Pure	5	V	Frequent	3/4	M	78	20	15/30	No	No
64	High AD Pure	5	V	Frequent	3/4	M	74	12	CDR 0	No	No
65	High AD Pure	5	V	Frequent	3/3	M	95	5	NA	No	No
66	High AD Pure	4	V	Frequent	3/4	M	77	5	21/30	No	No
67	High AD Pure	5	VI	Frequent	3/3	M	66	5	NA	No	No
68	High AD Pure	5	VI	Frequent	3/3	M	79	21	16/30	No	No
69	High AD Pure	5	VI	Frequent	3/4	M	63	2	NA	No	No
70	High AD Pure	5	VI	Frequent	3/3	M	83	7	NA	No	No
71	High AD Pure	5	VI	Frequent	3/4	M	59	7	14/30	No	No
72	High AD Pure	5	VI	Frequent	4/4	M	70	8	NA	No	No
51	High AD mixed	5	VI	Frequent	3/4	F	84	72	NA	diffuse neocortical	No
52	High AD mixed	5	V	Frequent	3/4	F	83	6	NA	diffuse neocortical	No
53	High AD mixed	5	VI	Frequent	4/4	F	75	NA	dementia reported	diffuse neocortical	stage 2
54	High AD mixed	4	V	Frequent	3/4	F	76	20	dementia reported	diffuse neocortical	stage 3
55	High AD mixed	5	V	Frequent	3/3	F	63	12	26/30	diffuse neocortical	stage 3
56	High AD mixed	5	V	Frequent	2/3	F	98	9	20/30	diffuse neocortical	stage 3
57	High AD mixed	5	VI	Frequent	4/4	F	75	8	22/30	amygdala-predominant	stage 1
58	High AD mixed	5	VI	Frequent	3/4	F	83	11	23/30	amygdala-predominant	no
59	High AD mixed	5	V	Frequent	3/3	F	88	4	2/30	amygdala-predominant	no
60	High AD mixed	5	VI	Frequent	4/4	F	76	3	14/30	limbic-transitional	stage 3
61	High AD mixed	5	V	Frequent	3/3	F	78	12	NA	limbic-transitional	stage 2
73	High AD mixed	5	V	Frequent	3/4	M	80	21	NA	diffuse neocortical	stage 2
74	High AD mixed	5	VI	Frequent	3/3	M	62	5	NA	diffuse neocortical	stage 2
75	High AD mixed	5	VI	Frequent	3/4	M	78	22	NA	diffuse neocortical	No
76	High AD mixed	5	V	Frequent	3/3	M	64	3	7/30	amygdala-predominant	No
77	High AD mixed	5	VI	Frequent	3/3	M	70	4	13/30	amygdala-predominant	stage 2
78	High AD mixed	5	VI	Frequent	3/3	M	87	9	NA	diffuse neocortical	No
79	High AD mixed	5	V	Frequent	3/3	M	94	144	NA	limbic-transitional	No
80	High AD mixed	5	VI	Frequent	4/4	M	91	14.25	NA	diffuse neocortical	stage 1
81	High AD mixed	5	V	Frequent	3/3	M	85	25	NA	amygdala-predominant	No
82	High AD mixed	5	V	Frequent	3/3	M	79	14	6/30	amygdala-predominant	stage 1
83	High AD mixed	5	VI	Frequent	3/3	M	83	11	NA	amygdala-predominant	stage 1

### Gallyas Silver impregnation and IHC staining

Gallyas Silver staining and immunohistochemistry (IHC) double stain were performed to quantify NP and non-NP. 8 μm thick sections of formalin-fixed, paraffin-embedded (FFPE) brain tissues were deparaffinized in xylene (2 × 5 min) and an ethanol series (100%, 100%, 90%, 70%) for 1 min each. Following that, a modified Gallyas Silver impregnation protocol was carried out as described in a method paper from the Saito lab [37].

Briefly, brain sections were placed in a Coplin jar filled with 5% periodic acid for 5 min, followed by two washes in dH_2_O for 5 min each. Sections were then immersed in an alkaline silver iodide solution for 1 min. After a few quick rinses with dH_2_O and a 10-min wash in 0.5% acetic acid, sections were placed in the developer solution (prepared 30 min prior and stored in a refrigerator at 4 °C) for 18–22 min. Following the development, sections were washed in 0.5% acetic acid for 3 min and then washed in dH_2_O for 5 min. Next, sections were placed in 0.1% gold chloride for 5 min, briefly rinsed in dH_2_O, and then incubated in 1% sodium thiosulphate solution for 5 min before being rinsed with tap water.

After completion of the silver stain, the IHC protocol was performed by incubating sections in a solution of 0.1M Tris (pH 7.6) and 0.05% Tween at high pressure for 15 min to induce antigen retrieval. This was followed by incubation of the sections in a PBS/H_2_O_2_ solution with 10% Triton-X for 20 min to quench endogenous peroxidases. After multiple washes with tap water and a 5-min wash in 0.1 M Tris (pH 7.6), sections were incubated in normal horse serum (Vector Labs) for 20 min, followed by blocking in 2% FBS/0.1 M Tris (pH 7.6) for 5 min. Anti-Aβ antibody, Ab5 (1:1000 dilution, Golde Lab, UF), was then diluted in a blocking buffer and incubated overnight at 4 °C. The following day, sections were rinsed in 0.1 M Tris (pH 7.6) and blocked in 2% FBS/0.1 M Tris (pH 7.6) for 5 min before incubating with the secondary antibody (anti-Mouse Alkaline Phosphatase ImmPRESS Polymer Reagent, Vector Labs) for 30 min. After a quick wash in 0.1 M Tris, color was developed using an Alkaline Phosphatase substrate kit (Vector Red Substrate Kit, AP- SK-5100) for 5 min. Then, sections were counterstained with hematoxylin (Sigma Aldrich, catalog# 51,275) for 1 min. Next, sections were rinsed in tap water and washed in isopropanol (2 × 5 min) before coverslipping with VectaMount Express mounting medium (Vector Laboratories catalog# ZK0209).

### Double IHC staining

Gallyas silver staining and p-tau are both used as markers of neuritic plaques. However, these two markers stain different phases of tau maturation. To reflect this, we also used anti-p-tau and anti-Aβ double immunolabeling to quantify the non-NP and NP in frontal cortex. For p-tau and Aβ plaques double staining, the IHC protocol was performed as previously described above with the following changes. After overnight incubation with both primary antibodies (anti-p-tau antibody, 7F2(1:5k dilution, B.Giasson Lab, UF)) and anti-Aβ antibody, D12B2(1:1000 dilution, Cell Signaling)), sections were first incubated in biotinylated secondary antibody (ImmPRESS-HRP Polymer anti-Rabbit Reagent) for 30 min and then developed with 3,3′-diaminobenzidine (Vector DAB, Vector Labs, catalog #51,275) for 1 min. Following a 10-min wash in tap water, sections were then immersed in an alkalinated secondary antibody (ImmPRESS-AP (Alkaline Phosphatase) Polymer anti-Mouse Reagent, Vector Labs, catalog# MP-5402) for 1 h and then developed using an Alkaline Phosphatase substrate kit (Vector Red Substrate Kit, AP- SK-5100) for 5 min.

### Spatial transcriptomics using the NanoString GeoMx Digital Spatial Profiling (DSP)

For GeoMx DSP study, total 12 representative frontal cortex brain samples were selected with 4 cases from each stages of ADNC (4 low AD cases, 4 intermediate AD cases, and 4 high AD cases). Three different 250 μm diameter ROIs (Non-NP ROI, NP ROI, and control no plaque ROI) were selected from each case. From each case, total 23 ROIs were selected which constitute 10 Non-NP ROIs, 10 NP ROIs, and 3 Control no plaque ROIs.

Nanostring GeoMx DSP experiments were performed according to the Nanostring GeoMx DSP Slide Preparation User Manual and GeoMx DSP NGS Readout User Manual obtained from NanoString University (university.nanostring.com). Briefly, 5 μm thick sections of formalin-fixed, paraffin-embedded (FFPE) brain tissues were deparaffinized and rehydrated, target retrieved, digested with proteinase K, and post-fixed with 10% Neutral-buffered formalin (NBF). Next, sections were incubated overnight with GeoMx Whole Transcriptomic Atlas (WTA)- Human RNA detection probes that are conjugated to DNA-oligonucleotides barcodes via a UV-photocleavable linker. Following a stringent wash, sections were stained with morphology and nuclear markers for Aβ plaques (Texas Red 594), p-tau 7F2 (Cy5 666), and DNA (SYTO-13) and imaged for visualization. 250 μm diameter ROIs were selected using NanoString GeoMx DSP, and then photocleaved oligonucleotides from each region-of-interest (ROI) were aspirated and collected into individual wells of a 96-well PCR collection plate. The collection plate was dried overnight, rehydrated to equal amounts, and then the library was prepared by PCR amplifying the oligo tags with PCR Master Mix and GeoMx SeqCode primers. After PCR, libraries were pooled and cleaned with AMPure XP beads (catalog# A63880) and assessed for quality and quantity using Qubit and Agilent BioAnalyzer DNA HS Kit. Finally, libraries were sequenced on an Illumina NovaSeq 6000 according to the manufacturer’s instructions. FASTQ files were processed into digital count conversion (DCC) files using NanoString GeoMx NGS Pipeline v2.0.21 on Illumina BaseSpace Sequence Hub.

### Data analysis

IHC-stained brain slides were code blinded using a random number generator (RANDOM.ORG) and then scanned using an Aperio AT2 slide scanner (Leica Biosystems). The blinded observer manually counted the NP and non-NP count using Aperio ImageScope 12.4.6 (Leica Biosystems). NP and non-NP counts were normalized to the total area of the tissue section.

For GeoMx WTA data analysis, raw counts were checked for quality control (QC) for both segment/ROI and biological probe. Segments with less than 1000 raw reads, less than 80% aligned reads, less than 50% sequencing saturation, and less than 20 nuclei count were removed. After segment and biological probe QC, a limit of quantification (LOQ) per segment and target was carried out to filter segments and targets that are expressed below the expression threshold. LOQ is a confidence threshold that is calculated based on the distribution of negative control probes. 5% LOQ was used for both segment and target as an expression threshold. Data were normalized to quartile 3 (Q3) for downstream data visualization and analysis. Q3 normalization allows similar gene expression ranges for all segments to reduce variance.

### Statistics

For IHC data quantification, one-way ANOVA with Tukey’s post-hoc multiple comparisons was used. For spatial transcriptomic analysis, linear mixed model (LMM) with Benjamini–Hochberg multiple test correction was used.

## Results

### Evidence for transformation of non-neuritic plaques into neuritic plaques during the progression of ADNC.

**Fig. 1:**
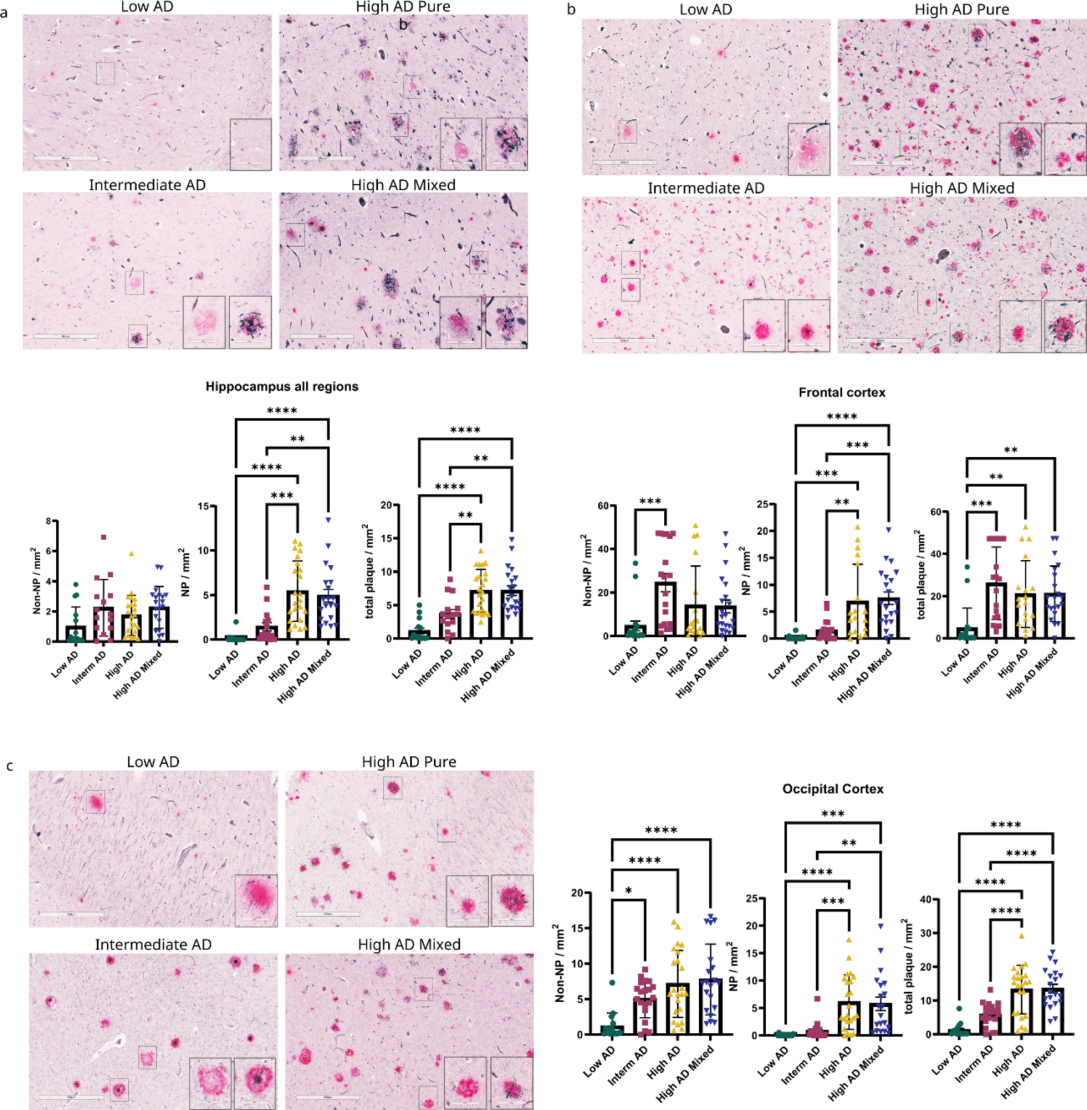
Non-NP are transformed into NP during the progression of ADNC. **a** Hippocampus all regions. CA1 region from different AD neuropathological changes were shown in low magnification (300 μm) and non-NP and NP from each case were shown, in insert, with high magnification (60 μm). NP and non-NP were determined by using modified Gallyas silver staining and anti-Aβ antibody Ab5 (anti-Mouse). Non-NP and NP count was manually counted by a blinded observer from the same case and compared between low AD (n = 15), intermediate AD (n = 15), high AD (n = 23), and high AD mixed pathology (n = 20). Between intermediate AD and high AD cases, non-NP is slightly lower in high AD but NP count is significantly higher in high AD. **b** Frontal cortex. Frontal cortex images from different AD neuropathological changes. Quantification of non-NP, NP, and total plaque (Non-NP and NP) were compared between low AD (n = 18), intermediate AD (n = 18), high AD (n = 19), and high AD mixed pathology (n = 21). Comparing intermediate AD and high AD cases, the non-NP count is lower in high AD, but NP is significantly increased in high AD. **c** Occipital Cortex. Occipital cortex images from different AD neuropathological changes- Low AD (n = 16), intermediate AD (n = 20), High AD (n = 23), and high AD mixed pathology (n = 20). No significant differences in non-NP between intermediate AD and high AD but NP is significantly increased in high AD.

To investigate the distribution of non-NP and NP and their relationship to each other, we selected 83 postmortem brain samples that were divided into four groups based on ADNC: “low AD”, “intermediate AD”, “high AD” (pure), and “high AD with mixed” pathology. For each case, we analyzed multiple brain regions and subregions to reflect the progression of ADNC. Hippocampal subregions and the inferior temporal gyrus were selected as a region affected early in ADNC progression. The frontal cortex and occipital cortex were included to interrogate brain regions increasingly affected in cases with ADNC intermediate and ADNC high respectively. To morphologically characterize and quantify non-NP and NP in the same tissue section, we stained 8 μm thick sections with Gallyas Silver staining and anti-Aβ antibody Ab5 [40]. For the purpose of our study, we defined NP by the presence of both Gallyas Silver positive DN and Ab5 positivity, whereas non-NP were defined by the presence of only anti- Aβ antibody Ab5 positivity. Although different Aβ plaque morphologies (e.g., diffuse, dense-core, etc.) were noted, all Aβ plaques within a given tissue sample were categorized as either non-NP or NP. Non-NP and NP count were manually counted by a blinded observer and then normalized to the total tissue area. As expected, total plaque counts and counts of Gallyas Silver + NP increased in the hippocampus, frontal cortex, and occipital cortex with increasing severity of ADNC from low AD to high AD (Fig. 1). Hippocampal subregions that are closer to the neocortex also show a significant increase in NP counts between intermediate AD and high AD (Additional file 1: Supplementary Fig. 1), while this trend was not as obvious in subregions of the hippocampus proper (Additional file 1: Supplementary Fig. 1). Counts of non-NP stagnated in the hippocampus, frontal cortex, and occipital cortex when comparing intermediate AD and high AD cases (Fig. 1). In general, we observed a divergence of counts for NP and non-NP between intermediate AD and high AD cases in all brain regions examined.

**Fig. 2:**
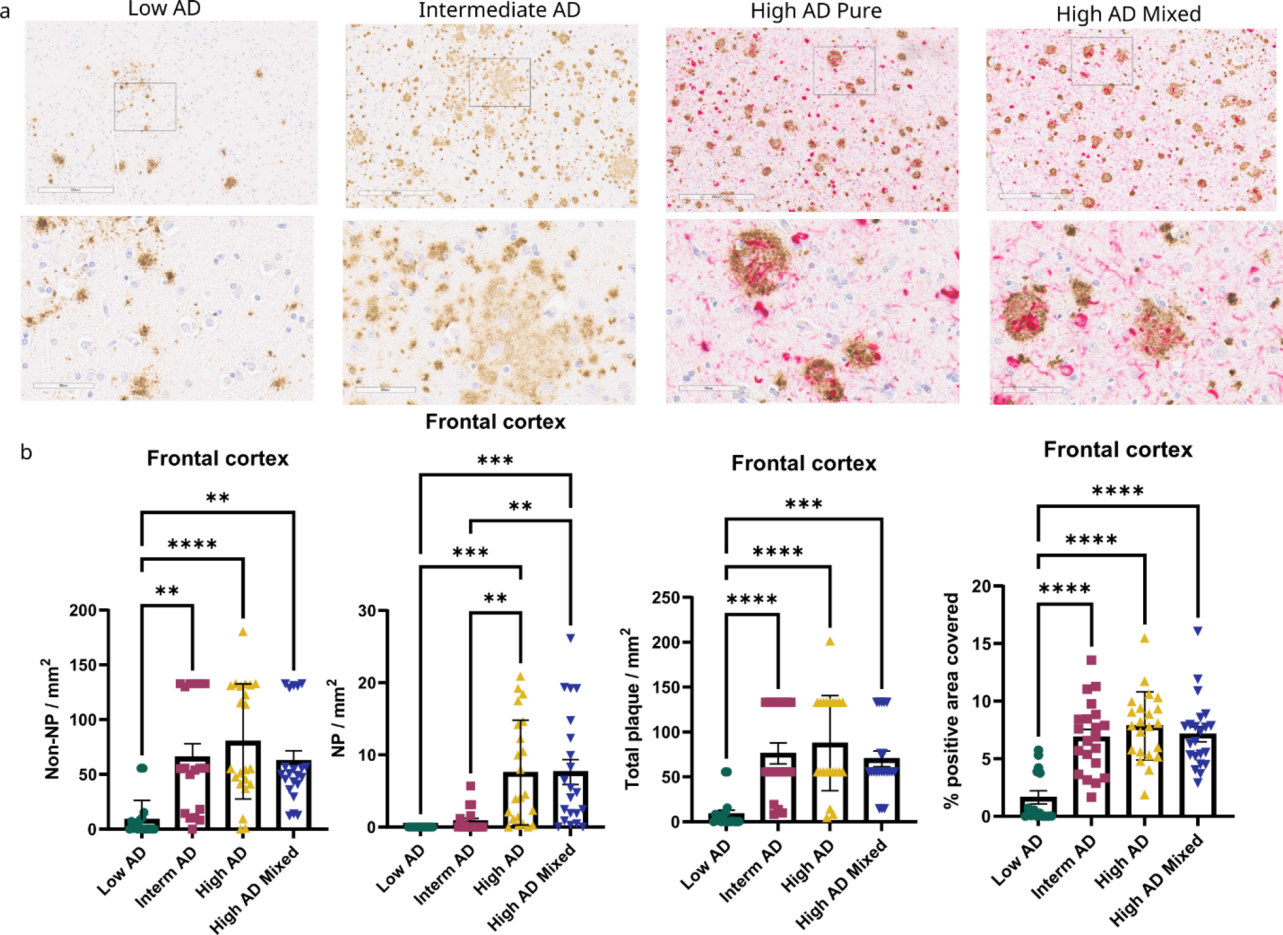
Non-NP are transformed into NP in the progression of ADNC using p-tau staining as a NP marker in the frontal cortex. **a** Low magnification (300 μm) and high magnification (60 μm) images of frontal cortex in different AD neuropathological changes. Non-NP, NP, and total plaque count were compared between low AD (n = 18), intermediate AD (n = 17), high AD (n = 23), and high AD mixed pathology (n = 21). NP and non-NP were determined by using p-tau (7F2) staining and anti-Aβ antibody D12B2 (anti-Rabbit). **b** Comparing intermediate AD and high AD, the non-NP count is not changed, but the NP count is significantly higher in high AD cases. The manual count of total plaques shares a similar trend with the software (Qupath) quantification of the percent positivity of amyloid plaque from consecutive tissues

Both phosphorylated tau (p-tau) antibody staining and Gallyas silver staining are currently used to visualize NP [50, 72]. However, Gallyas silver staining and p-tau antibodies stain tau aggregates at different maturation phases [49]. To test the correlation between Gallyas Silver + NP and accumulation of p-tau around Aβ deposition, we next performed p-tau (antibody 7F2 (Thr205) [75] and Aβ double labeling in the frontal cortex. For this analysis, we defined Aβ plaques stained by anti- Aβ antibody, D12B2 surrounded by neuritic 7F2 immunoreactivity as NP, and D12B2 + Aβ plaques without neuritic 7F2 staining as non-NP. This analysis revealed an increase in 7F2 + NP during the progression of ADNC, whereas the count of non-NP was not significantly different between intermediate AD and high AD cases (Fig. 2). These data support the notion derived from our analysis of Gallyas Silver + NP and suggest that 7F2 + NP counts trends are increasing in a similar fashion during the progression of ADNC.

### NFT appear to precede NP in the hippocampus but follow NP in cortical regions

**Fig. 3:**
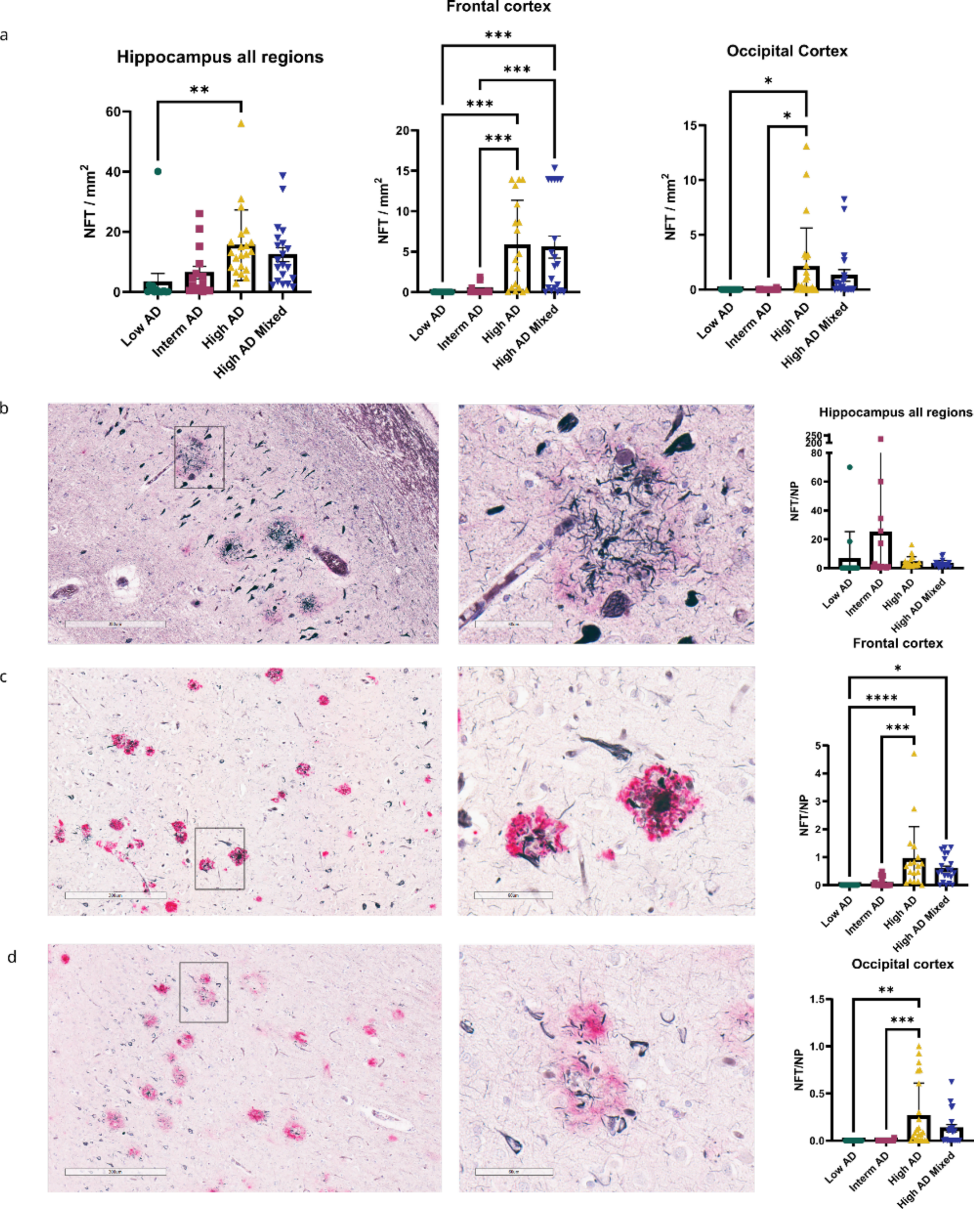
NFT appear to precede NP in hippocampus but follow NP in cortical regions. **a** Quantification of NFT count (NFT/mm^2^) in hippocampus all regions, frontal cortex, and occipital cortex. NFT count, along with non-NP and NP count is quantified from the same sample using modified Gallyas silver staining and anti-A β antibody Ab5 (anti-Mouse). NFT count is increased in the progression of AD neuropathological changes with a significant increase from low AD and intermediate AD to high AD cases. Sample size- low AD (n = 14), intermediate AD (n = 15), high AD (n = 21), and high AD mixed pathology (n = 20). **b** NFT/NP ratio in the hippocampus all regions. CA1 region (Low magnification 300 μm) from a high AD case with NP and NFT depicted in high magnification (60 μm). No significant differences in NFT/NP in the hippocampus all regions between AD stages. Sample size- low AD (n = 14), intermediate AD (n = 15), high AD (n = 21), and high AD mixed pathology (n = 19). **c** NFT/NP ratio in frontal cortex. Frontal cortex (Low magnification 300 μm) from a High AD case with NP and NFT depicted in high magnification (60 μm). No significant differences in NFT/NP between low AD and intermediate AD, but significant increase in NFT/NP from low AD and intermediate AD to high AD. Sample size- low AD (n = 18), intermediate AD (n = 18), high AD (n = 19) and high AD mixed pathology (n = 21). **d** NFT/NP ratio in occipital cortex. Occipital cortex (Low magnification 300 μm) from a high AD case with NP and NFT depicted in high magnification (60 μm). NFT/NP ratio is significantly increased from low AD and intermediate AD to high AD. Sample size- low AD (n = 16), intermediate AD (n = 19), high AD (n = 23), and high AD mixed pathology (n = 20)

NP are suggested to be a driving force for cortical tau aggregation [30]. To elucidate the relationship between NP and NFT in ADNC progression, we examined the spatiotemporal distribution of NP and NFT. Gallyas Silver impregnation and IHC staining enable the visualization of both NP and NFT in the same brain section. As expected, NFT counts increased in the hippocampus, frontal cortex, and occipital cortex when comparing low AD and intermediate AD cases (Fig. 3a). A significant increase of NFT counts was also seen in cortical regions (frontal cortex and occipital cortex) during the progression from ADNC from intermediate AD to high AD while NFT counts in the hippocampal subregions did not follow this trend (Fig. 3a; Additional file 1: Supplementary Fig. 2). NFT counts from individual hippocampal subregions show a significant increase comparing low AD to high AD, but no significant differences in NFT counts are observed between intermediate AD and high AD cases (Additional file 1: Supplementary Fig. 2). This is contrary to NP counts, which significantly increased when comparing intermediate AD to high AD in almost all brain regions examined (Fig. 1) except for a few hippocampal subregions (dentate gyrus, CA4, CA3, entorhinal cortex, and subiculum) (Additional file 1: Supplementary Fig. 1). To get a better understanding of the relationship between NP and NFT during the progression of ADNC, we calculated the ratio of NFT counts and NP counts for a given brain region. An increase in the NFT/NP ratio during the progression of ADNC from intermediate AD to high AD would support the “seeding” of NFT by p-tau + NP. This analysis revealed interesting brain region-specific characteristics of the NFT/NP ratio. In the hippocampus, we observed no significant differences in the NFT/NP ratio during the progression of ADNC from intermediate AD to high AD (Fig. 3b). The NFT/NP ratio in hippocampal subregions also showed no significant differences between ADNC groups, except for an increase from low AD to high AD in CA4 (Additional file 1: Supplementary Fig. 2b). There was, however, a significant increase in the NFT/NP ratio in the frontal cortex and occipital cortex, comparing intermediate AD to high AD cases (Fig. 3c, d). In summary, we observed that relative abundance of NFT in relation to NP is increased in cortical regions in the progression of ADNC, but this relationship is not observed in hippocampus.

### Neuronal systems, calcium-dependent events, and Neurotransmission across chemical synapse pathways are upregulated around NP compared to non-NP

**Fig. 4:**
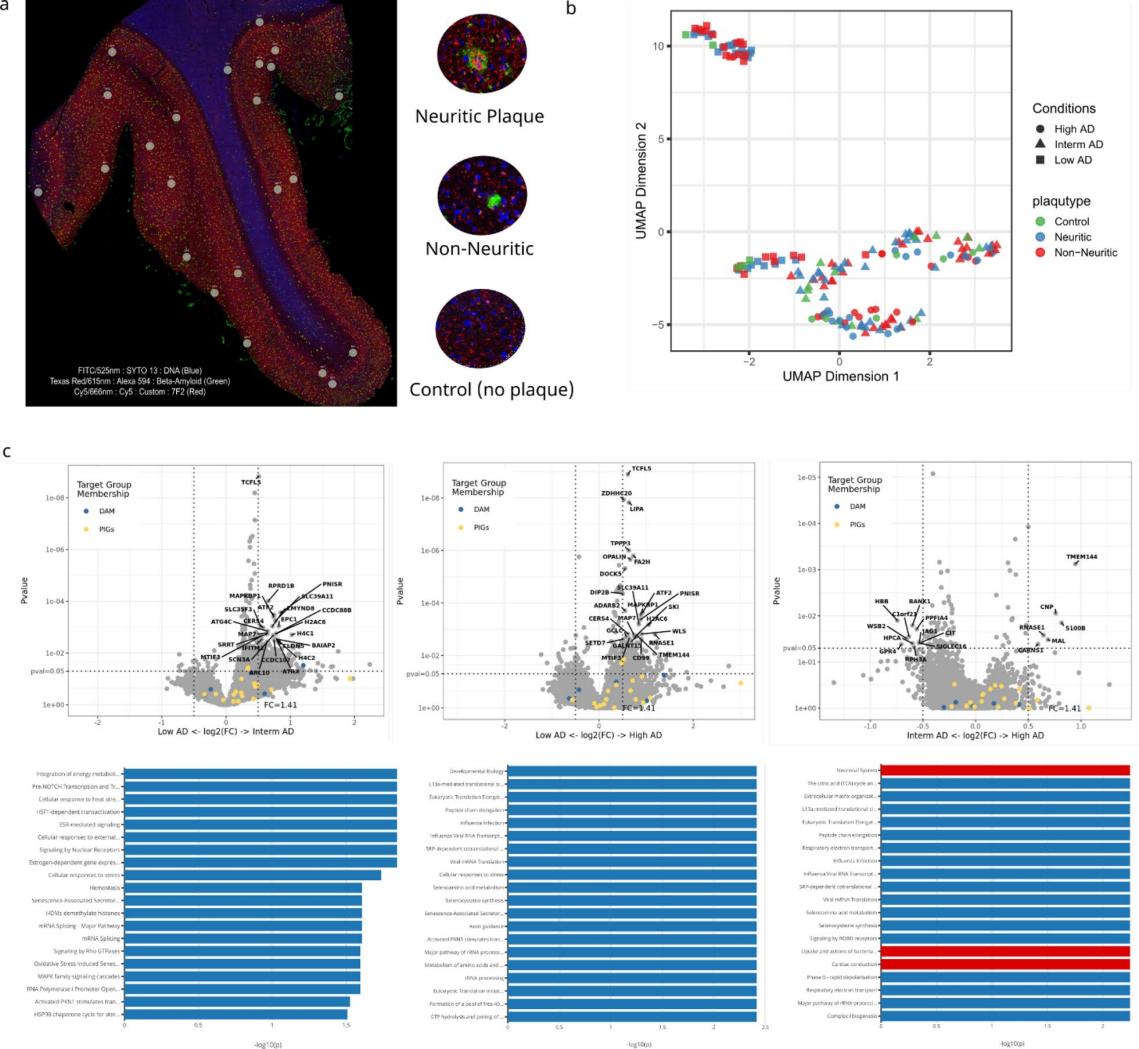
Spatial transcriptomic changes between different stages of ADNC. **a** Frontal cortex stained for amyloid plaque (green) and p-tau (red). NP and non-NP is determined by anti-tau antibody 7F2 positivity around the amyloid plaque. Amyloid plaque (green) with 7F2 positivity is considered NP. Amyloid plaque without 7F2 positivity is considered non-NP. Control is ROI without amyloid plaque. A total of 23 250- μm diameter ROI (10 NP, 10 non-NP, and 3 control) were selected per sample. 12 brain samples – low AD (n = 4), intermediate AD (n = 4) and high AD (n = 4) were used. However, 1 low AD case and 2 high AD cases failed to pass the quality control for analysis. **b** UMAP dimension reduction of spatial transcriptomic changes in different ADNC and plaque types. The biggest transcriptomic changes are seen between stages of ADNC. **c** Volcano plot and pathway analysis of different stages of ADNC – Low AD vs. Intermediate AD, Low AD vs. High AD, and Intermediate AD vs. High AD. *Left panel*: Volcano plot and pathway analysis showing spatial transcriptomic differences between Low AD and Intermediate AD cases. Top 20 pathways that are upregulated in both Low AD and Intermediate AD microenvironment. The blue color represents pathways that are upregulated in Intermediate AD or downregulated in Low AD. The red color represents pathways that are upregulated in Low AD or downregulated in Intermediate AD. Integration of energy metabolism (adj p-value = 0.014), Cellular response to heat stress (adj p-value = 0.014), and Hemostasis (adj p-value = 0.024) are upregulated in Intermediate AD. *Middle panel*: Volcano plot and pathway analysis showing spatial transcriptomic differences between Low AD and High AD. Top 20 pathways that are upregulated in both Low AD and High AD microenvironments. The blue color represents pathways that are upregulated in High AD or downregulated in Low AD. The red color represents pathways that are upregulated in Low AD or downregulated in High AD. Developmental Biology (adj p-value = 0.003), Cellular response to stress (adj p-value = 0.003), and Axon guidance pathways (adj p-value = 0.003), are upregulated in High AD. *Right panel*: Volcano plot and pathway analysis showing spatial transcriptomic differences between Intermediate AD and High AD. Top 20 pathways that are upregulated in both Intermediate AD and High AD microenvironment. The blue color represents pathways that are upregulated in High AD or downregulated in Intermediate AD. The red color represents pathways that are upregulated in Intermediate AD or downregulated in High AD. Extracellular matrix organization (adj p-value = 0.005) is upregulated in High AD while, neuronal system pathway (adj p-value = 0.005), is upregulated in Intermediate AD

Our histological data suggest that non-NP are transformed into NP in the progression of ADNC, and NP tau might initiate the cortical spreading of NFT. To better understand the differences in the tissue microenvironment between non-NP and NP, we employed a spatial transcriptomics approach using the human whole transcriptome atlas (WTA) on the NanoString GeoMx Digital Spatial Profiler (DSP) platform. For this analysis, we selected 12 representative cases from our histological study (4 low AD cases, 4 intermediate AD cases, and 4 high AD cases). For each sample, we selected 23 ROI (10 NP ROI, 10 Non-NP ROI, and 3 Control (no plaque) ROI) (Fig. 4a). Non-NP were defined by the presence of only Aβ staining (green, Fig. 4a, right panel, middle image), NP were defined by the presence of both Aβ and 7F2 p-tau staining (red and green, Fig. 4a, right panel, top image), and Control (no plaque) ROI was defined as an area with no Aβ staining and no significant 7F2 staining (Fig. 4a, right panel, bottom image). 2 high AD cases and 1 low AD case failed the quality control assessment and were removed from the data analysis. The remaining cases were analyzed using the NanoString GeoMx DSP Analysis Suite (Version 2.4.0.421).

First, we analyzed gene expression differences between different stages of ADNC. For this, spatial transcriptomics data from 3 low AD cases (23 ROI per case), 4 Intermediate AD cases (23 ROI per case), and 2 high AD cases (23 ROI per case) were compared to each other using a linear mixed model (LMM) to account for the sampling of multiple ROI segments per tissue. UMAP, a dimension reduction technique, was used to identify general patterns in the data. UMAP data showed that low AD cases have significantly different gene expression compared to intermediate AD cases and high AD cases (Fig. 4b). The top 25 differentially expressed genes (DEG) in each comparison of ADNC are highlighted in the volcano plot (Fig. 4c). Compared to Low AD cases, intermediate AD cases exhibited upregulation in pathways such as integration of energy metabolism, cellular response to heat stress, and hemostasis. On the other hand, high AD cases showed increased activation of pathways related to developmental biology, cellular response to stress, and axon guidance. (Fig. 4c). When comparing intermediate AD and high AD cases, the extracellular matrix organization pathway is most upregulated in high AD cases, whereas the neuronal systems pathway exhibits the most profound downregulation in high AD cases compared to intermediate AD cases (Fig. 4c). Overall, these comparisons demonstrate that hemostasis, cellular response to stress, and neuronal system pathways are disrupted with increased severity of ADNC from low AD to high AD.

**Fig. 5:**
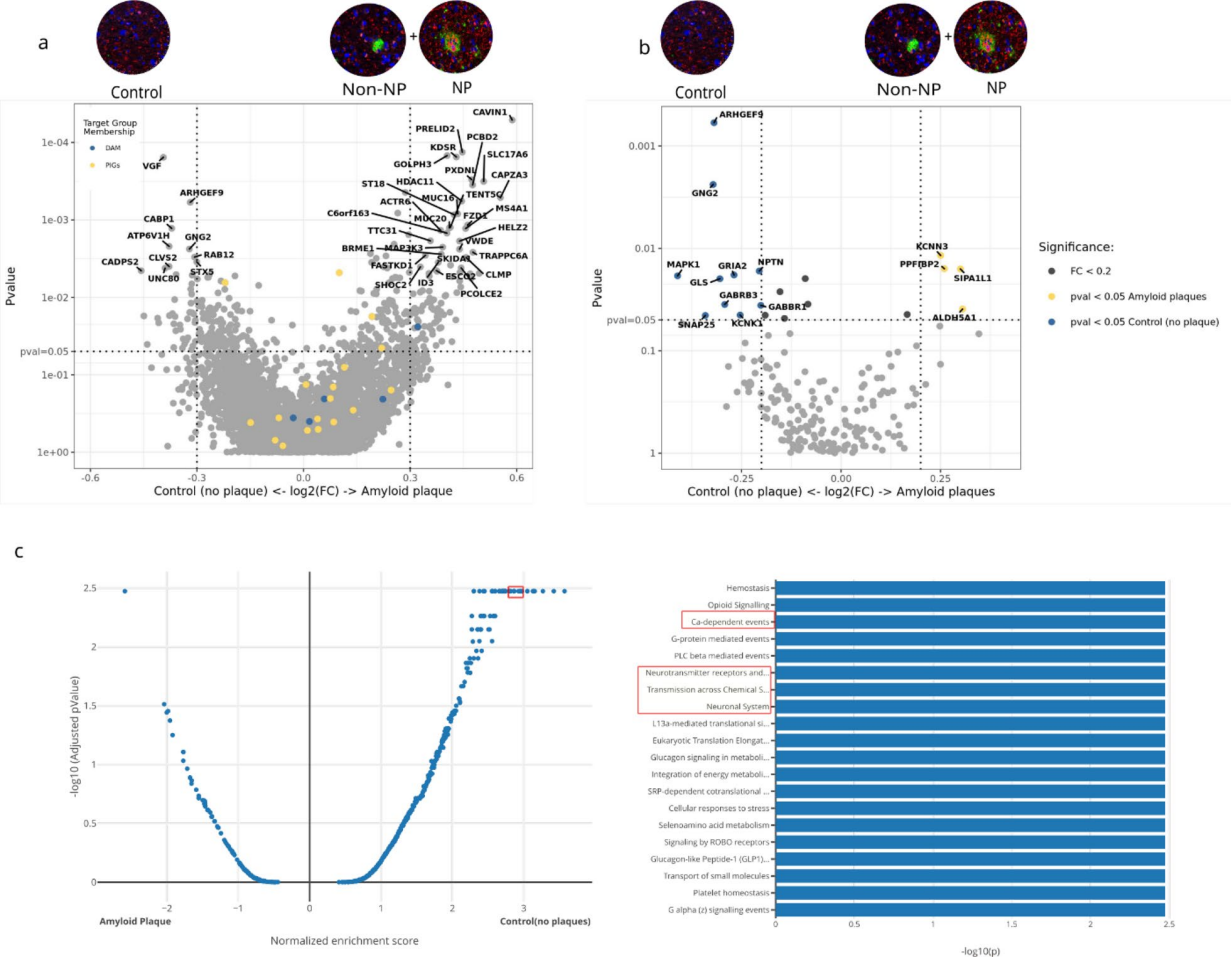
Spatial transcriptomic analysis shows neuronal system and Ca-dependent event pathway is downregulated in amyloid plaque microenvironment. **a** Gene expression differences between control (no plaque microenvironment) and amyloid plaque (NP + non-NP) in volcano plot. Top 25 DEGs in both microenvironments that are above the set threshold (P-value = 0.05, log2 FC = 0.3) are highlighted/labelled. Plaque-induced Genes (PIGs) and Disease associated microglia (DAM) genes are more distributed towards amyloid plaque microenvironment. **b** Neuronal system related gene expression differences between control (no plaque microenvironment) and amyloid plaque (NP + non-NP) in volcano plot. Neuronal system related genes are downregulated in amyloid plaque microenvironment compared to control (no plaque) microenvironment. Top DEGs are labelled. P-value = 0.05, log2 FC = 0.2. **c** Top 20 Pathways of control (no plaque) and amyloid plaque microenvironment in postmortem brain sample. The blue color represents pathways that are upregulated in Control (no plaque) or downregulated in amyloid plaque microenvironment. Neuronal system (adj p-value = 0.003), Ca-dependent events (adj p-value = 0.003) and Transmission across Chemical Synapses pathway (adj p-value = 0.003) are more downregulated in amyloid plaque microenvironment

Next, we compared the spatial transcriptomic difference between the amyloid plaque microenvironment (NP ROI + non-NP ROI) and the control (no plaque) microenvironment (Control ROI) across all cases. DEG demonstrated several highly upregulated genes in the plaque microenvironment compared to control, including caveolin-1(CAVIN1) [25], Histone Deacetylase 11 (HDAC11), Frizzled class receptor 1 (FZD1), and membrane-spanning 4-domains A1 (MS4A1) [42] among others (Fig. 5a). Among genes that were downregulated in the plaque microenvironment are VGF [3], Calcium-dependent activator protein for secretion (CADPS2), and G Protein Subunit Gamma 2 (GNG2) (Fig. 5a). Of particular interest, CAVIN1, a cholesterol-binding membrane protein, and VGF, a nerve growth factor, are shown to play an important role in AD pathogenesis [3, 25]. Top DEG in the amyloid plaque and control (no plaque) microenvironment are highlighted in the volcano plot (Fig. 5a) and also shown in the Additional file 1: Table 4. Pathway analysis between the control (no plaque) microenvironment and Aβ plaque microenvironment (Non-NP and NP combined) revealed a significant downregulation of neuronal systems (adj p-value = 0.003), Ca-dependent event (adj p-value = 0.003), and transmission across chemical synapses (adj p-value = 0.003) in the Aβ plaque microenvironment (Fig. 5c). This is consistent with prior studies showing the depletion of neuronal markers around Aβ plaques [80]. Bart De Strooper and colleagues showed that Aβ plaques induce expression of a set of genes, which they termed Plaque-induced genes (PIGs) [13] and a previous study by Keren-Shaul and colleagues has established a gene expression signature of microglia around Aβ plaques, termed Disease-associated microglia (DAM) [34]. We examined the distribution of PIG and DAM in our sample and found that most of the PIG and DAM genes are more distributed within the Aβ plaque microenvironment as compared to the control (no plaque) microenvironment (Fig. 5a). DAM and PIG lists are derived from the following papers [13, 34, 46].

**Fig. 6:**
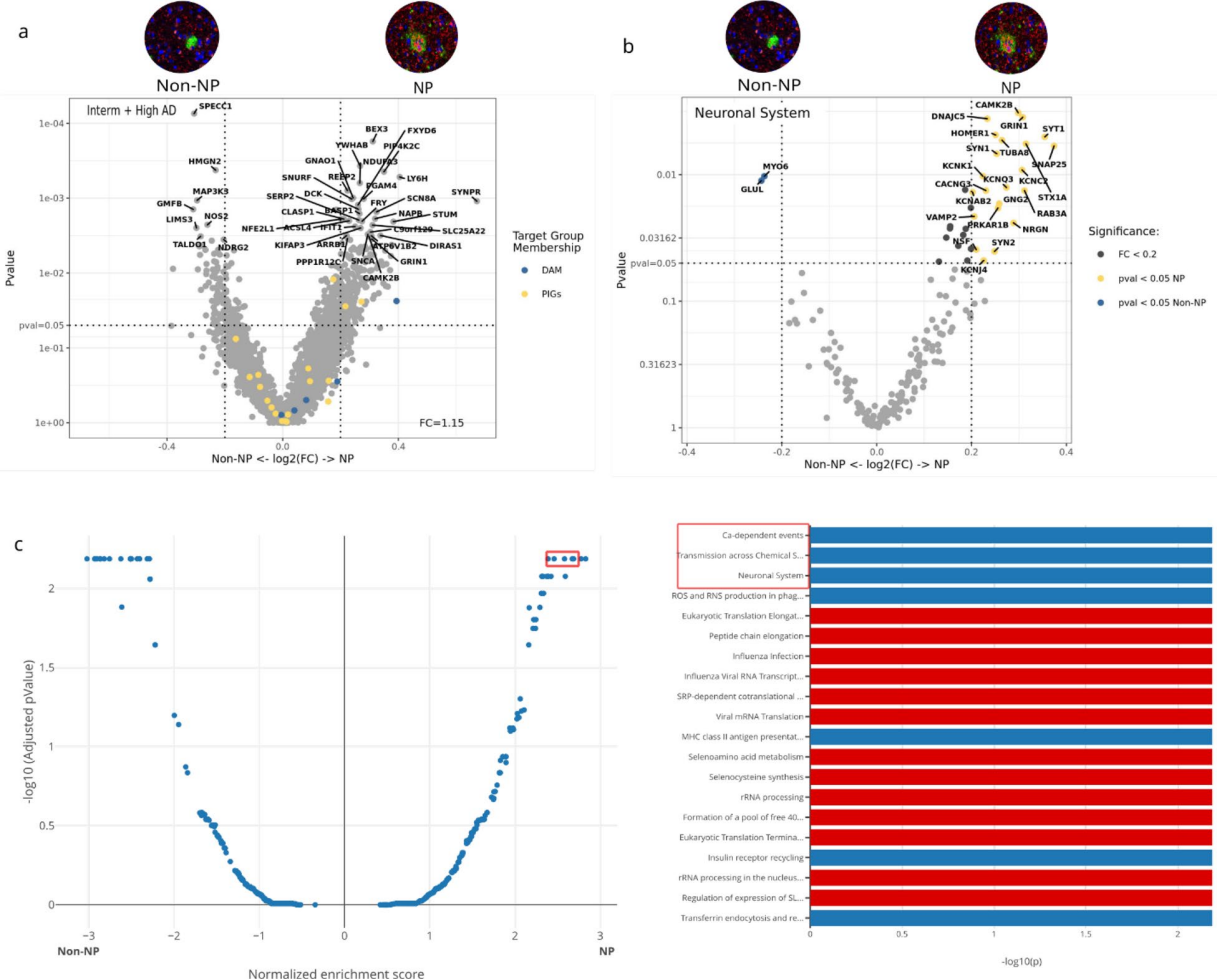
Between non-NP and NP, neuronal system, transmission across chemical synapse, and Ca-dependent event pathway are upregulated in NP and downregulated in non-NP microenvironment. **a** Volcano plot shows non-NP and NP spatial transcriptomic differences. Low AD cases lack p-tau + NP and removed from the analysis. SYNPR is most upregulated in NP microenvironment suggesting NP are pre-synaptic and axonal in origin. Top 25 DEGs in both non-NP and NP microenvironments that are above the set threshold (P-value = 0.05, log2 FC = 0.2) are highlighted/labelled. **b** Volcano plot shows genes involved in neuronal system are more downregulated in non-NP microenvironment. GLUL and MYO6 were upregulated in non-NP and SNAP25 and SYT1 were upregulated in NP. Top DEGs are labelled. **c** Top 20 pathways that are upregulated in both non-NP and NP microenvironment. The blue color represents pathways that are upregulated in NP or downregulated in Non-NP. The red color represents pathway that are upregulated in Non-NP. Pathway analysis shows that Ca-dependent events (adj p-value = 0.006), Transmission across Chemical Synapses (adj p-value = 0.006), and Neuronal system (adj p-value = 0.006) are upregulated in NP and downregulated in non-NP

Lastly, we compared the spatial transcriptomic changes between the NP and the non-NP microenvironment. For this analysis of gene expression differences between NP and non-NP, low AD cases were excluded due to lack of p-tau + NP. Prominent among the highly upregulated genes in the NP environment are synaptoporin (SYNPR), lymphocyte antigen 6 family member H (LY6H) [74], and brain abundant membrane attached signal protein 1 (Basp1) [14]. The upregulation of the SYNPR gene in the NP microenvironment suggests that NP are pre-synaptic and axonal in origin (Fig. 6a). Top DEG in non-NP and NP microenvironments are highlighted/labeled in the plot (Fig. 6a) and also shown in Additional file 1: Table 5. PIG genes are equally distributed between non-NP and NP, but DAM markers are more distributed within NP microenvironment (Fig. 6a). Pathway analysis demonstrated that neuronal systems (adj p-value = 0.006), transmission across chemical synapses (adj p-value = 0.006), Ca-dependent event (adj p-value = 0.006), and MHC class II antigen presentation (adj p-value = 0.006) were significantly upregulated in the NP microenvironment compared to the non-NP microenvironment (Fig. 6c). Eukaryotic translation elongation (adj p-value = 0.006), influenza infection (adj p-value = 0.006), and viral mRNA translation (adj p-value = 0.006) were pathways showing downregulation in the NP microenvironment compared to the non-NP microenvironment (Fig. 6c). A volcano plot highlighting genes in the neuronal systems pathway shows that most of the neuronal genes, such as synaptosome- associated protein 25 (SNAP25), synaptotagmin 1 (SYT1), and others were more upregulated around NP, while a few neuronal genes, such as myosin VI (MYO6) and glutamine synthase (GLUL) were downregulated around NP (Fig. 6b).

When comparing the control (no plaque) with non-neuritic and neuritic microenvironments, neuronal system, Ca-dependent events, neurotransmission across chemical synapses, opioid signaling, etc. were downregulated in the non-neuritic microenvironment, while those pathways were not altered in the neuritic plaque microenvironment (Additional file 1: Supplementary Fig. 3). The DEG and pathway comparison of non-NP and NP with control (no plaque) microenvironment is shown in Additional file 1: Supplementary Fig. 3.

**Fig. 7:**
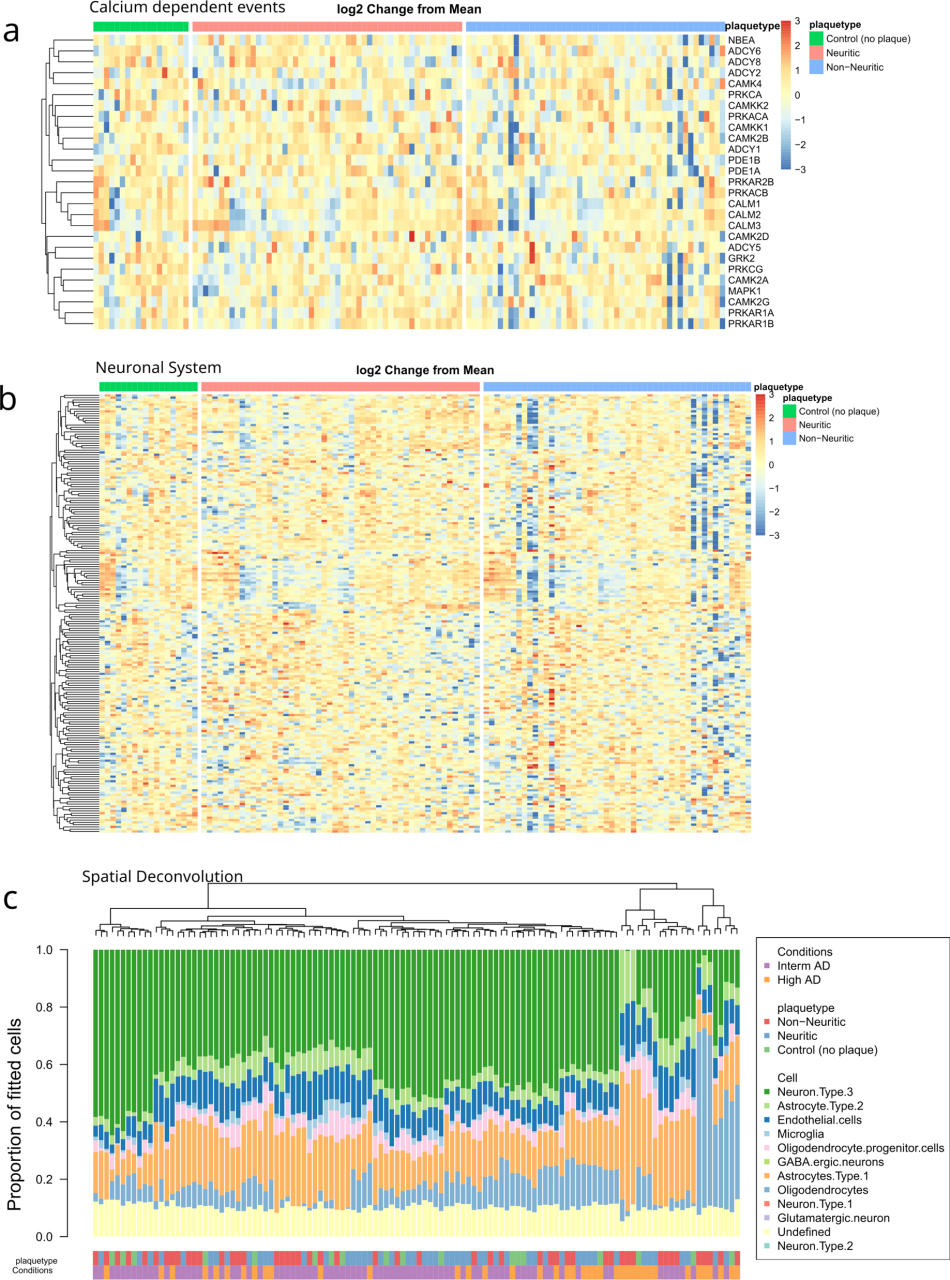
Comparison of transcriptomic pathways between control, non-NP and NP. **a** Calcium-dependent events and **b** neuronal system related gene heatmap from NanoString DSP compared between control (no plaque) microenvironment, NP microenvironment, and non-NP microenvironment. Log2 change from mean shows non-NP microenvironment is most downregulated in both Calcium dependent genes and neuronal system genes, followed by NP microenvironment and then control. Sample size (n) = 6. **c** Spatial Decon algorithm derived from the NanoString DSP shows cell type proportion in control, NP and non-NP microenvironment

When comparing and ranking gene expression changes in control (no plaque), the non-NP, and NP microenvironments, the non-NP microenvironment shows the most pronounced downregulation of neuronal system and Ca-dependent event pathways, followed by the NP microenvironment, and then the control (no plaque) microenvironment (Fig. 6a, b). These data may provide evidence for the hypothesis that DN around NP are a compensatory and regenerative mechanism to the neuronal injury posed by amyloid plaque. The failure to compensate and regenerate the neurons around amyloid plaque might be contributing to the formation of NP (Fig. 7).

Although NanoString GeoMx (Whole Transcriptome Atlas) DSP cannot reach single-cell level resolution, GeoMx WTA data can be integrated with single-cell data to predict the proportion of cell types. To achieve this, transcripts within the microenvironment (ROI) were deconvoluted into estimated mixed cell types using the NanoString human brain cell-profile library derived from their scRNA-seq experiment (GitHub—Nanostring-Biostats/SpatialDecon). Spatial Deconvolution data showed that neuronal cells were relatively less abundant around the non-NP microenvironment compared to the NP microenvironment (Fig. 6c).

## Discussion

In this study, we used postmortem human brain tissue samples from the hippocampus, frontal cortex, and occipital cortex to assess the distribution of non-NP and NP in the progression of ADNC. Our data suggest that non-NP are being transformed into NP in the progression of ADNC, and NP tau might initiate the cortical spreading of NFT. Additionally, our spatial transcriptomic analysis indicated that the NP microenvironment shows more profound upregulation of neuronal system and Ca-dependent events than non-NP, suggesting that NP might be a failed regeneration attempt and a compensatory mechanism.

Prior studies have examined the distribution of diffuse plaques and dense-cored plaques in AD progression, but this is the first study that assesses the distribution of non-NP and NP during the progression of ADNC. In general, the non-NP count increased from low AD to intermediate AD and then stagnated in high AD cases, whereas the NP count increased from low AD over intermediate AD to high AD. The total plaque count (non-NP + NP) increased from low AD to intermediate AD and then plateaued. These data are suggestive of the notion that non-NP are transformed into NP during the progression of ADNC and that this transformation is able to trigger cortical NFT formation (Additional file 1: Supplementary Fig. 4). Although our study is cross-sectional and descriptive in nature, there is substantial evidence of non-neuritic to neuritic plaque transformation in animal studies using two-photon microscopy for live in vivo imaging. In these studies the appearance of amyloid plaque deposition led to the selective formation of neuritic dystrophy [7, 10]. Interestingly, neuritic dystrophies were highly plastic, and some neuritic dystrophies can disappear or return to normalcy during the two-photon imaging window [7]. It has been shown that DN form sequentially in different layers in 5xFAD mice [64], an animal model of amyloid deposition; initial layers are formed by proteins related to autophagy and lysosomal function, while outer layers are formed by the endoplasmic reticulum and late endosome related proteins [64, 65]. In general, APP, lamp1, and ubiquitin immunoreactive DN appear early in AD progression, while tau immunoreactive DN appear late [5, 6, 20, 22, 67, 78]. This temporal sequence of DN formation might suggest that early DN could be highly plastic and reversible, while late DN could be irreversible in AD progression. Future studies employing model systems that allow for tracking of individual Aβ plaque over time should investigate the precise mechanism of the transformation of non-NP into NP using different DN markers such as APP, Lamp1, ubiquitin, and p-tau.

Although two-photon imaging studies in animal models discussed above demonstrated non-neuritic to neuritic plaque transformation, other animal studies argued for independent evolvement of different Aβ morphology subtypes. The inoculation of distinct Aβ strains or conformers into host transgenic models showed that different Aβ morphology subtypes arise from different populations of Aβ strains or conformers [48, 76, 77]. These studies favor the idea that distinct Aβ strains compete and dominate each other during the progression of AD, rather than the transformation of one Aβ morphology subtype over another.

There are a few hypotheses about DN/NP formation. The three major hypotheses are that (1) extracellular Aβ causes axonal dystrophy, (2) intracellular Aβ causes dystrophic neurites through autophagy and lysosomal disruption, and (3) p-tau + droplet degeneration-mediated ferroptosis leads to NP formation. The first hypothesis is based on Aβ oligomer studies suggesting the exogenous application of Aβ oligomers in cell culture models resulted in beaded neurites with disruption in microtubules and axonal transport [2, 57, 61]. The second hypothesis postulates that Aβ /APP fragments accumulate in the lysosome and autophagic vesicles, and the failure of autolysosomal digestion of Aβ/APP fragments distorts the plasma membrane, forming flower-like neuritic dystrophy [17, 38, 55]. The third hypothesis proposed that iron overload in the brain leads to ferroptosis, a type of cell death due to iron overload. Ferroptosis causes the release of p-tau and iron into the extracellular space, which is encased by Aβ deposition (as a protective mechanism), leading to neuritic dystrophy [41, 66]. Our study didn’t address the mechanistic underpinnings of DN/NP formation. However, we observed that DN in the hippocampus shows a different morphology than in cortical regions. Taken together with our finding that the NP/NFT ratio follows distinct trajectories in the hippocampus and cortical regions, it is tempting to speculate that different mechanisms of DN/NP formation (hypothesis 1,2,3) could occur simultaneously or sequentially in the same or different brain regions.

Another critical, yet unanswered, question in AD pathophysiology is how Aβ plaques interact with or trigger neuronal tauopathy. Studies over the years have suggested that Aβ plaques synergistically accelerate and increase tau aggregation and propagation [1, 4, 51, 58]. However, the mechanisms behind this proposed Aβ plaque-tau interaction are still unknown. One possible mechanism that links Aβ plaques and tau aggregation is DN/NP formation [61]. Prior animal model studies suggest that Aβ plaque facilitates tau accumulation by translocating hyperphosphorylated tau in axonal dystrophy to the soma, forming NFT [30]. Our data support the notion of brain region- specific differences in NP tau mediated facilitation of NFT formation. In hippocampal subregions and the entorhinal cortex, NP tau does not appear to facilitate NFT formation; instead, NFT are formed before NP in the progression of ADNC. Ghoshal et al. examined tau conformational changes in 37 clinically staged cases, and suggest that NFT precedes the appearance of NP in hippocampal regions [26]. On the contrary, NP formation preceded NFT formation in cortical regions, such as the frontal cortex and occipital cortex, suggesting that NP might be initiating the cortical spread of NFT. A previous neuroimaging study suggested that regional Aβ plaque-tau interaction in the ITG promoted the onset and acceleration of tau spreading [39]. Therefore, NP might be the pivotal factor that drives the spread of tau in cortical regions after local Aβ plaque and tau interaction in the ITG. Although highly speculative, we propose that age-related hippocampal/medial temporal lobe (MTL) tau inclusions are a prerequisite for AD pathogenesis, such that Aβ plaques enable the spread of hippocampus/MTL tau inclusions into the cortical regions via DN. In the absence of age-related hippocampus/MTL tau inclusions, Aβ plaques fail to trigger tauopathy in cortical regions and patients hence developing resilience. This is evident in pathological aging, where elderly subjects with abundant Aβ plaque pathology but no significant NFT pathology do not show obvious cognitive decline [19]. It has been shown that cortical NFT and NP have a higher correlation with cognitive decline and are more associated with the clinical picture of AD [52, 53]. Therefore, DN /NP might be the key interface between Aβ plaque and tau that exerts a clinical picture of AD.

Our spatial transcriptomic data show that the Aβ plaque microenvironment exhibits a downregulation of neuronal system and Ca-dependent events compared to the control microenvironment. Previous studies have demonstrated that dendrites, axons, and neuronal markers are less abundant around Aβ plaques [35, 80], thus suggesting Aβ plaques as space-consuming entities that can create a localized “mass effect” [73]. However, the exact reason for the lack of neuronal markers around Aβ plaques is a matter of intense discussion. For example, Aβ is suggested to be toxic to neurons in many in vitro experiments [33, 63], but these experiments used high concentrations of Aβ that are probably not physiologically relevant.

Interestingly, among morphological subtypes of Aβ plaques, the non-NP microenvironment showed the most profound downregulation of neuronal system and Ca-dependent events pathways compared to NP and control microenvironments. Our data provide evidence to speculate that the DN around NP might be a regenerative attempt by neurons whose regenerative failure exacerbates the axonal transport and mislocalized axonal proteins, such as APP and tau. The view that DN formation represents the result of regenerative failure has been previously described [18, 73]. In general, central nervous system (CNS) axons have limited regeneration capacity compared to peripheral nervous system (PNS). During CNS axonal injury, microglia, astrocytes, and other immune cells infiltrate the lesion site and release extracellular matrix components (ECM) that form a glial scar [71]. Inhibitory ECMs can limit axonal growth and thus restrict CNS regenerative processes [11, 24]. Although our data suggest a regenerative failure, we only examined transcriptomic changes, and it will be important to correlate our findings with changes in the proteome, as transcriptomic changes do not always correlate with proteomic changes, which has major implications for mechanistic interpretations of the data. The observed upregulation of neuronal systems and Ca-dependent events pathways around NP could represent a compensatory mechanism, whereby the loss of neurons around NP triggers upregulation of related transcripts to compensate for the loss.

Neuroinflammation is another important facet of AD, along with Aβ plaques and NFT. Microglia activation is the most significant mediator of neuroinflammation in AD [59, 60]. The exact role of microglia either as neuroprotective or detrimental in AD pathophysiology is still elusive. Single-cell RNA seq and spatial transcriptomic studies of the AD mouse model showed that certain microglia genes are highly expressed/upregulated around amyloid plaques [34, 80]. However, those studies didn’t differentiate between different morphological subtypes of Aβ plaques such as diffuse plaques, dense-core plaques, and neuritic plaques. Prior studies showed that microglia are more clustered and activated around NP compared to diffuse non-neuritic plaque [28, 44, 47, 54]. As expected, our study showed that DAM genes are more distributed around the NP microenvironment compared to non-NP. SPP1 is the most upregulated DAM gene in the NP microenvironment. Pathway analysis of non-NP and NP showed that MHC class II antigen presentation was significantly upregulated in the NP microenvironment compared to the non-NP microenvironment. These data support the notion that activated microglia are intimately associated with the transformation of non-NP to NP.

There are several limitations of our study. Our study is correlational and the progression of ADNC is extrapolated from different individual brains at different time points. To truly observe the proposed transformation of non-NP into NP, a single Aβ plaque would have to be tracked in vivo over a long period of time. The sample size for our spatial transcriptomic study is low due to technical limitations, but our data correlate well with available whole-tissue and single-cell transcriptomic studies. Our study only examined the transcriptomic changes in the control, non-NP and NP microenvironment. Future studies comparing transcriptomic and proteomic changes will be required to paint a more holistic picture of the local microenvironment around different morphological subtypes of Aβ plaques.

### Supplementary Information

**Additional file 1**. Supplementary figures

## Abbreviations

AD: Alzheimer’s disease
NP: Neuritic Plaques
APP: Amyloid precursor protein
ECM: Extracellular matrix component
ACH: Amyloid cascade hypothesis
DN: Dystrophic Neurites
ADNC: Alzheimer’s Disease Neuropathological changes
DSP: Digital spatial profiling
PA: Pathological aging
PART: Primary aged-related Tauopathy
PSEN: Presenilin
WTA: Whole transcriptomic atlas
NFT: Neurofibrillary tangles
LOQ: Limit of quantification
IHC: Immunohistochemistry
LMM: Linear mixed model
PIG: Plaque induced gene
DAM: Disease associated microglia
ITG: Inferior temporal gyrus

## References

[1] Aragão-Gomes L, Andrea-Hipp S, Rijal-Upadhaya A, Balakrishnan K, Ospitalieri S, Koper MJ, Largo-Barrientos P, Uytterhoeven V, Reichwald J, Rabe S, Vandenberghe R, Giudici C, Willem M, Staufenbiel M, Rudolf-Thal D (2023). Aβ-induced acceleration of Alzheimer-related τ-pathology spreading and its association with prion protein. Acta Neuropathol.

[2] Bassil R, Shields K, Granger K, Zein I, Ng S, Chih B (2021). Improved modeling of human AD with an automated culturing platform for iPSC neurons, astrocytes and microglia. Nat Commun.

[3] Beckmann ND, Lin W-J, Wang M, Cohain AT, Charney AW, Wang P, Ma W, Wang Y-C, Jiang C, Audrain M, Comella PH, Fakira AK, Hariharan SP, Belbin GM, Girdhar K, Levey AI, Seyfried NT, Dammer EB, Tu Z, Ehrlich ME, Zhang B, Salton SR, Schadt EE (2020). Multiscale causal networks identify VGF as a key regulator of Alzheimer’s disease. Nat Commun.

[4] Bennett RE, Devos SL, Dujardin S, Corjuc B, Gor R, Gonzalez J, Roe AD, Frosch MP, Pitstick R, Carlson GA, Hyman BT (2017). Enhanced Tau aggregation in the presence of amyloid β. Am J Pathol.

[5] Benzing WC, Brady DR, Mufson EJ, Armstrong DM (1993). Evidence that transmitter-containing dystrophic neurites precede those containing paired helical filaments within senile plaques in the entorhinal cortex of nondemented elderly and Alzheimer’s disease patients. Brain Res.

[6] Blanchard V, Moussaoui S, Czech C, Touchet N, Bonici B, Planche M, Canton T, Jedidi I, Gohin M, Wirths O, Bayer TA, Langui D, Duyckaerts C, Tremp G, Pradier L (2003). Time sequence of maturation of dystrophic neurites associated with Aβ deposits in APP/PS1 transgenic mice. Exp Neurol.

[7] Blazquez-Llorca L, Valero-Freitag S, Rodrigues EF, Merchán-Pérez Á, Rodríguez JR, Dorostkar MM, DeFelipe J, Herms J (2017). High plasticity of axonal pathology in Alzheimer’s disease mouse models. Acta Neuropathol Commun.

[8] Boon BDC, Bulk M, Jonker AJ, Morrema THJ, van den Berg E, Popovic M, Walter J, Kumar S, van der Lee SJ, Holstege H, Zhu X, Van Nostrand WE, Natté R, van der Weerd L, Bouwman FH, van de Berg WDJ, Rozemuller AJM, Hoozemans JJM (2020). The coarse-grained plaque: a divergent Aβ plaque-type in early-onset Alzheimer’s disease. Acta Neuropathol.

[9] Boutajangout A, Authelet M, Blanchard V, Touchet N, Tremp G, Pradier L, Brion J-P (2004). Characterisation of cytoskeletal abnormalities in mice transgenic for wild-type human tau and familial Alzheimer’s disease mutants of APP and presenilin-1. Neurobiol Dis.

[10] Brendza RP, Bacskai BJ, Cirrito JR, Simmons KA, Skoch JM, Klunk WE, Mathis CA, Bales KR, Paul SM, Hyman BT, Holtzman DM (2005). Anti-Abeta antibody treatment promotes the rapid recovery of amyloid-associated neuritic dystrophy in PDAPP transgenic mice. J Clin Invest.

[11] Busch SA, Silver J (2007). The role of extracellular matrix in CNS regeneration This review comes from a themed issue on Development Edited by Ben Barres and Mu-Ming Poo. Curr Opin Neurobiol.

[12] Cai Y, Xiong K, Zhang X-M, Cai H, Luo X-G, Feng J-C, Clough RW, Struble RG, Patrylo PR, Chu Y, Kordower JH, Yan X-X (2010). β-Secretase-1 elevation in aged monkey and Alzheimer’s disease human cerebral cortex occurs around the vasculature in partnership with multisystem axon terminal pathogenesis and β-amyloid accumulation. Eur J Neurosci.

[13] Chen WT, Lu A, Craessaerts K, Pavie B, Sala Frigerio C, Corthout N, Qian X, Laláková J, Kühnemund M, Voytyuk I, Wolfs L, Mancuso R, Salta E, Balusu S, Snellinx A, Munck S, Jurek A, Fernandez Navarro J, Saido TC, Huitinga I, Lundeberg J, Fiers M, De Strooper B (2020). Spatial transcriptomics and in situ sequencing to study Alzheimer’s Disease. Cell.

[14] Chung D, Shum A, Caraveo G (2020). GAP-43 and BASP1 in Axon regeneration: implications for the treatment of neurodegenerative diseases. Front Cell Dev Biol.

[15] Cras P, Kawai M, Lowery D, Gonzalez-DeWhitt P, Greenberg B, Perry G (1991). Senile plaque neurites in Alzheimer disease accumulate amyloid precursor protein. Proc Natl Acad Sci U S A.

[16] Crook R, Verkkoniemi A, Perez-Tur J, Mehta N, Baker M, Houlden H, Farrer M, Hutton M, Lincoln S, Hardy J, Gwinn K, Somer M, Paetau A, Kalimo H, Ylikoski R, Pöyhönen M, Kucera S, Haltia M (1998). A variant of Alzheimer’s disease with spastic paraparesis and unusual plaques due to deletion of exon 9 of presenilin 1. Nat Med.

[17] D’Andrea MR, Nagele RG, Wang H-Y, Peterson PA, Lee DHS (2001). Evidence that neurones accumulating amyloid can undergo lysis to form amyloid plaques in Alzheimer’s disease. Histopathology.

[18] Dewitt DA, Silver J (1996). Regenerative failure: a potential mechanism for neuritic dystrophy in Alzheimer’s disease. Exp Neurol.

[19] Dickson DW, Crystal HA, Mattiace LA, Masur DM, Blau AD, Davies P, Yen S-H, Aronson MK (1992). Identification of normal and pathological aging in prospectively studied nondemented elderly humans. Neurobiol Aging.

[20] Dickson TC, King CE, McCormack GH, Vickers JC (1999). Neurochemical diversity of dystrophic neurites in the early and late stages of Alzheimer’s Disease. Exp Neurol.

[21] Dickson TC, Vickers JC (2001). The morphological phenotype of β-amyloid plaques and associated neuritic changes in Alzheimer’s disease. Neuroscience.

[22] Duyckaerts C, Delaère P, Poulain V, Brion J-P, Hauw J-J (1988). Does amyloid precede paired helical filaments in the senile plaque? A study of 15 cases with graded intellectual status in aging and Alzheimer disease. Neurosci Lett.

[23] Fiala JC, Feinberg M, Peters A, Barbas H (2007). Mitochondrial degeneration in dystrophic neurites of senile plaques may lead to extracellular deposition of fine filaments. Brain Struct Funct.

[24] Fitch MT, Silver J (2008). CNS injury, glial scars, and inflammation: Inhibitory extracellular matrices and regeneration failure. Exp Neurol.

[25] Gaudreault SB, Dea D, Poirier J (2004). Increased caveolin-1 expression in Alzheimer’s disease brain. Neurobiol Aging.

[26] Ghoshal N, García-Sierra F, Wuu J, Leurgans S, Bennett DA, Berry RW, Binder LI (2002). Tau Conformational Changes Correspond to Impairments of Episodic Memory in Mild Cognitive Impairment and Alzheimer’s Disease. Exp Neurol.

[27] Gowrishankar S, Yuan P, Wu Y, Schrag M, Paradise S, Grutzendler J, De Camilli P, Ferguson SM (2015). Massive accumulation of luminal protease-deficient axonal lysosomes at Alzheimer’s disease amyloid plaques. Proc Natl Acad Sci U S A.

[28] Haga S, Akai K, Ishii T (1989). Demonstration of microglial cells in and around senile (neuritic) plaques in the Alzheimer brain. Acta Neuropathol.

[29] Haroutunian V (1998). Regional distribution of neuritic plaques in the nondemented elderly and subjects with very mild alzheimer disease. Arch Neurol.

[30] He Z, Guo JL, McBride JD, Narasimhan S, Kim H, Changolkar L, Zhang B, Gathagan RJ, Yue C, Dengler C, Stieber A, Nitla M, Coulter DA, Abel T, Brunden KR, Trojanowski JQ, Lee VMY (2018). Amyloid-β plaques enhance Alzheimer’s brain tau-seeded pathologies by facilitating neuritic plaque tau aggregation. Nat Med.

[31] Hyman BT, Phelps CH, Beach TG, Bigio EH, Cairns NJ, Carrillo MC, Dickson DW, Duyckaerts C, Frosch MP, Masliah E, Mirra SS, Nelson PT, Schneider JA, Thal R, Thies B, Trojanowski JQ, Vinters H V, Montine TJ (2012) National Institute on Aging’s Alzheimer; Association guidelines for the neuropathologic assessment of Alzheimer’s disease; National Institute on Aging; Alzheimer’s Association guidelines for the neuropathologic assessment of Alzheimer’s disease. doi: 10.1016/j.jalz.2011.10.007

[32] Ichimata S, Martinez-Valbuena I, Forrest SL, Kovacs GG (2022). Expanding the spectrum of amyloid-β plaque pathology: the Down syndrome associated ‘bird-nest plaque’. Acta Neuropathol.

[33] Jin M, Shepardson N, Yang T, Chen G, Walsh D, Selkoe DJ (2011). Soluble amyloid beta-protein dimers isolated from Alzheimer cortex directly induce Tau hyperphosphorylation and neuritic degeneration. Proc Natl Acad Sci U S A.

[34] Keren-Shaul H, Spinrad A, Weiner A, Matcovitch-Natan O, Dvir-Szternfeld R, Ulland TK, David E, Baruch K, Lara-Astaiso D, Toth B, Itzkovitz S, Colonna M, Schwartz M, Amit I (2017). A unique microglia type associated with restricting development of Alzheimer’s Disease. Cell.

[35] Knowles RB, Wyart C, Buldyrev SV, Cruz L, Urbanc B, Hasselmo ME, Stanley HE, Hyman BT (1999). Plaque-induced neurite abnormalities: implications for disruption of neural networks in Alzheimer’s disease. Proc Natl Acad Sci U S A.

[36] Kuchibhotla KV, Goldman ST, Lattarulo CR, Wu H-Y, Hyman BT, Bacskai BJ (2008). Abeta plaques lead to aberrant regulation of calcium homeostasis in vivo resulting in structural and functional disruption of neuronal networks. Neuron.

[37] Kuninaka N, Kawaguchi M, Ogawa M, Sato A, Arima K, Murayama S, Saito Y (2015). Simplification of the modified Gallyas method. Neuropathology.

[38] Lee J-H, Yang D-S, Goulbourne CN, Im E, Stavrides P, Pensalfini A, Chan H, Bouchet-Marquis C, Bleiwas C, Berg MJ, Huo C, Peddy J, Pawlik M, Levy E, Rao M, Staufenbiel M, Nixon RA (2022). Faulty autolysosome acidification in Alzheimer’s disease mouse models induces autophagic build-up of Aβ in neurons, yielding senile plaques. Nat Neurosci.

[39] Lee WJ, Brown JA, Kim HR, La Joie R, Cho H, Lyoo CH, Rabinovici GD, Seong JK, Seeley WW (2022). Regional Aβ-tau interactions promote onset and acceleration of Alzheimer’s disease tau spreading. Neuron.

[40] Levites Y, Das P, Price RW, Rochette MJ, Kostura LA, McGowan EM, Murphy MP, Golde TE (2006). Anti-Aβ42- and anti-Aβ40-specific mAbs attenuate amyloid deposition in an Alzheimer disease mouse model. J Clin Investig.

[41] Lopes KO, Sparks DL, Streit WJ (2008). Microglial dystrophy in the aged and Alzheimer’s disease brain is associated with ferritin immunoreactivity. Glia.

[42] Ma J, Yu J-T, Tan L MS4A Cluster in Alzheimer’s Disease. doi: 10.1007/s12035-014-8800-z 10.1007/s12035-014-8800-z 24981432

[43] Mabrouk R, Miettinen PO, Tanila H (2023). Most dystrophic neurites in the common 5xFAD Alzheimer mouse model originate from axon terminals. Neurobiol Dis.

[44] Mackenzie IRA, Hao C, Munoz DG (1995). Role of microglia in senile plaque formation. Neurobiol Aging.

[45] Malek-Ahmadi M, Perez SE, Chen K, Mufson EJ (2016). Neuritic and Diffuse Plaque Associations with Memory in Non-Cognitively Impaired Elderly. J Alzheimers Dis.

[46] McFarland KN, Chakrabarty P (2022). Microglia in Alzheimer’s Disease: a Key Player in the Transition Between Homeostasis and Pathogenesis. Neurotherapeutics.

[47] McGeer PL, Itagaki S, Tago H, McGeer EG (1987). Reactive microglia in patients with senile dementia of the Alzheimer type are positive for the histocompatibility glycoprotein HLA-DR. Neurosci Lett.

[48] Meyer-Luehmann M, Coomaraswamy J, Bolmont T, Kaeser S, Schaefer C, Kilger E, Neuenschwander A, Abramowski D, Frey P, Jaton AL, Vigouret JM, Paganetti P, Walsh DM, Mathews PM, Ghiso J, Staufenbiel M, Walker LC, Jucker M (2006). Exogenous induction of cerebral beta-amyloidogenesis is governed by agent and host. Science.

[49] Moloney CM, Lowe VJ, Murray ME (2021). Visualization of neurofibrillary tangle maturity in Alzheimer’s disease: A clinicopathologic perspective for biomarker research. Alzheimers Dement.

[50] Montine TJ, Phelps CH, Beach TG, Bigio EH, Cairns NJ, Dickson DW, Duyckaerts C, Frosch MP, Masliah E, Mirra SS, Nelson PT, Schneider JA, Thal DR, Trojanowski JQ, Vinters HV, Hyman BT (2012). National Institute on Aging-Alzheimer’s Association guidelines for the neuropathologic assessment of Alzheimer’s disease: a practical approach. Acta Neuropathol.

[51] Musiek ES, Holtzman DM (2015). Three Dimensions of the Amyloid Hypothesis: Time, Space, and “Wingmen”. Nat Neurosci.

[52] Nelson PT, Abner EL, Schmitt FA, Kryscio RJ, Jicha GA, Smith CD, Davis DG, Poduska JW, Patel E, Mendiondo MS, Markesbery WR (2010). Modeling the association between 43 different clinical and pathological variables and the severity of cognitive impairment in a large autopsy cohort of elderly persons. Brain Pathol.

[53] Nelson PT, Alafuzoff I, Bigio EH, Bouras C, Braak H, Cairns NJ, Castellani RJ, Crain BJ, Davies P, Del Tredici K, Duyckaerts C, Frosch MP, Haroutunian V, Hof PR, Hulette CM, Hyman BT, Iwatsubo T, Jellinger KA, Jicha GA, Kövari E, Kukull WA, Leverenz JB, Love S, Mackenzie IR, Mann DM, Masliah E, McKee AC, Montine TJ, Morris JC, Schneider JA, Sonnen JA, Thal DR, Trojanowski JQ, Troncoso JC, Wisniewski T, Woltjer RL, Beach TG (2012). Correlation of Alzheimer disease neuropathologic changes with cognitive status: a review of the literature. J Neuropathol Exp Neurol.

[54] Ohgami T, Kitamoto T, Shin RW, Kaneko Y, Ogomori K, Tateishi J (1991). Increased senile plaques without microglia in Alzheimer’s disease. Acta Neuropathol.

[55] Pensalfini A, Albay R, Rasool S, Wu JW, Hatami A, Arai H, Margol L, Milton S, Poon WW, Corrada MM, Kawas CH, Glabe CG (2014). Intracellular amyloid and the neuronal origin of Alzheimer neuritic plaques. Neurobiol Dis.

[56] Phinney AL, Deller T, Stalder M, Calhoun ME, Frotscher M, Sommer B, Staufenbiel M, Jucker M (1999). Cerebral amyloid induces aberrant axonal sprouting and ectopic terminal formation in amyloid precursor protein transgenic mice. J Neurosci.

[57] Pike CJ, Cummings BJ, Cotman CW (1992). β-Amyloid induces neuritic dystrophy in vitro. NeuroReport.

[58] Pooler AM, Polydoro M, Maury EA, Nicholls SB, Reddy SM, Wegmann S, William C, Saqran L, Cagsal-Getkin O, Pitstick R, Beier DR, Carlson GA, Spires-Jones TL, Hyman BT (2015). Amyloid accelerates tau propagation and toxicity in a model of early Alzheimer’s disease.

[59] Prokop S, Miller KR, Heppner FL (2013). Microglia actions in Alzheimer’s disease. Acta Neuropathol.

[60] Prokop S, Miller KR, Labra SR, Pitkin RM, Hoxha K, Narasimhan S, Changolkar L, Rosenbloom A, Lee VMY, Trojanowski JQ (2019). Impact of TREM2 risk variants on brain region-specific immune activation and plaque microenvironment in Alzheimer’s disease patient brain samples. Acta Neuropathol.

[61] Sadleir KR, Kandalepas PC, Buggia-Prévot V, Nicholson DA, Thinakaran G, Vassar R (2016). Presynaptic dystrophic neurites surrounding amyloid plaques are sites of microtubule disruption, BACE1 elevation, and increased Aβ generation in Alzheimer’s disease. Acta Neuropathol.

[62] Selkoe DJ, Hardy J (2016). The amyloid hypothesis of Alzheimer’s disease at 25 years. EMBO Mol Med.

[63] Shankar GM, Li S, Mehta TH, Garcia-Munoz A, Shepardson NE, Smith I, Brett FM, Farrell MA, Rowan MJ, Lemere CA, Regan CM, Walsh DM, Sabatini BL, Selkoe DJ (2008). Amyloid-beta protein dimers isolated directly from Alzheimer’s brains impair synaptic plasticity and memory. Nat Med.

[64] Sharoar MG, Hu X, Ma XM, Zhu X, Yan R (2019). Sequential formation of different layers of dystrophic neurites in Alzheimer’s brains. Mol Psychiatry.

[65] Sharoar MG, Palko S, Ge Y, Saido TC, Yan R (2021). Accumulation of saposin in dystrophic neurites is linked to impaired lysosomal functions in Alzheimer’s disease brains. Mol Neurodegener.

[66] Streit WJ, Rotter J, Winter K, Müller W, Khoshbouei H, Bechmann I (2022). Droplet Degeneration of Hippocampal and Cortical Neurons Signifies the Beginning of Neuritic Plaque Formation. Journal of Alzheimer’s Disease.

[67] Su JH, Cummings BJ, Cotman CW (1993). Identification and distribution of axonal dystrophic neurites in Alzheimer’s disease. Brain Res.

[68] Supnet C, Bezprozvanny I (2010). The dysregulation of intracellular calcium in Alzheimer disease. Cell Calcium.

[69] Tanzi RE (2012). The genetics of Alzheimer disease. Cold Spring Harb Perspect Med.

[70] Trejo-Lopez JA, Yachnis AT, Prokop S (2022). Neuropathology of Alzheimer’s Disease. Neurotherapeutics.

[71] Varadarajan SG, Hunyara JL, Hamilton NR, Kolodkin AL, Huberman AD Leading Edge Central nervous system regeneration. doi: 10.1016/j.cell.2021.10.029 10.1016/j.cell.2021.10.029 PMC10896592 34995518

[72] Woodhouse A, Vickers JC, Adlard PA, Dickson TC (2009). Dystrophic neurites in TgCRND8 and Tg2576 mice mimic human pathological brain aging. Neurobiol Aging.

[73] Woodhouse A, West AK, Chuckowree JA, Vickers JC, Dickson TC (2005). Does beta-amyloid plaque formation cause structural injury to neuronal processes?. Neurotox Res.

[74] Wu M, Liu CZ, Barrall EA, Rissman RA, Joiner WJ (2021). Unbalanced Regulation of α7 nAChRs by Ly6h and NACHO Contributes to Neurotoxicity in Alzheimer’s Disease. J Neurosci.

[75] Xia Y, Xia Y, Prokop S, Prokop S, Prokop S, Prokop S, Gorion KMM, Gorion KMM, Kim JD, Kim JD, Sorrentino ZA, Sorrentino ZA, Bell BM, Bell BM, Manaois AN, Manaois AN, Chakrabarty P, Chakrabarty P, Chakrabarty P, Davies P, Giasson BI, Giasson BI, Giasson BI (2020) Tau Ser208 phosphorylation promotes aggregation and reveals neuropathologic diversity in Alzheimer’s disease and other tauopathies. Acta Neuropathol Commun 8. doi: 10.1186/S40478-020-00967-W 10.1186/s40478-020-00967-w PMC7310041 32571418

[76] Xu G, Fromholt S, Borchelt DR (2022) Modeling the Competition between Misfolded Aβ Conformers That Produce Distinct Types of Amyloid Pathology in Alzheimer’s Disease. Biomolecules 12. doi: 10.3390/biom12070886 10.3390/biom12070886 PMC9313290 35883442

[77] Xu G, Fromholt SE, Chakrabarty P, Zhu F, Liu X, Pace MC, Koh J, Golde TE, Levites Y, Lewis J, Borchelt DR (2020) Diversity in Aβ deposit morphology and secondary proteome insolubility across models of Alzheimer-type amyloidosis. Acta Neuropathol Commun 8. doi: 10.1186/S40478-020-00911-Y 10.1186/s40478-020-00911-y PMC7137436 32252825

[78] Yasuhara O, Kawamata T, Aimi Y, McGeer EG, McGeer PL (1994). Two types of dystrophic neurites in senile plaques of alzheimer disease and elderly non-demented cases. Neurosci Lett.

[79] Yuan P, Zhang M, Tong L, Morse TM, McDougal RA, Ding H, Chan D, Cai Y, Grutzendler J (2022). PLD3 affects axonal spheroids and network defects in Alzheimer’s disease. Nature.

[80] Zeng H, Huang J, Zhou H, Meilandt WJ, Dejanovic B, Zhou Y, Bohlen CJ, Lee SH, Ren J, Liu A, Tang Z, Sheng H, Liu J, Sheng M, Wang X (2023). Integrative in situ mapping of single-cell transcriptional states and tissue histopathology in a mouse model of Alzheimer’s disease. Nat Neurosci.

