# Supplementary material for: Transformation of non-neuritic into neuritic plaques during AD progression drives cortical spread of tau pathology via regenerative failure
Source: Acta Neuropathol Commun. 2023 Dec 1;11:190. doi: 10.1186/s40478-023-01688-6 (PMC10691154; doi:10.1186/s40478-023-01688-6)
Supplement: Supplementary file 1 — Additional file 1. Supplementary figures [file 40478_2023_1688_MOESM1_ESM.docx]

**SUPPLEMENTARY FIGURES**


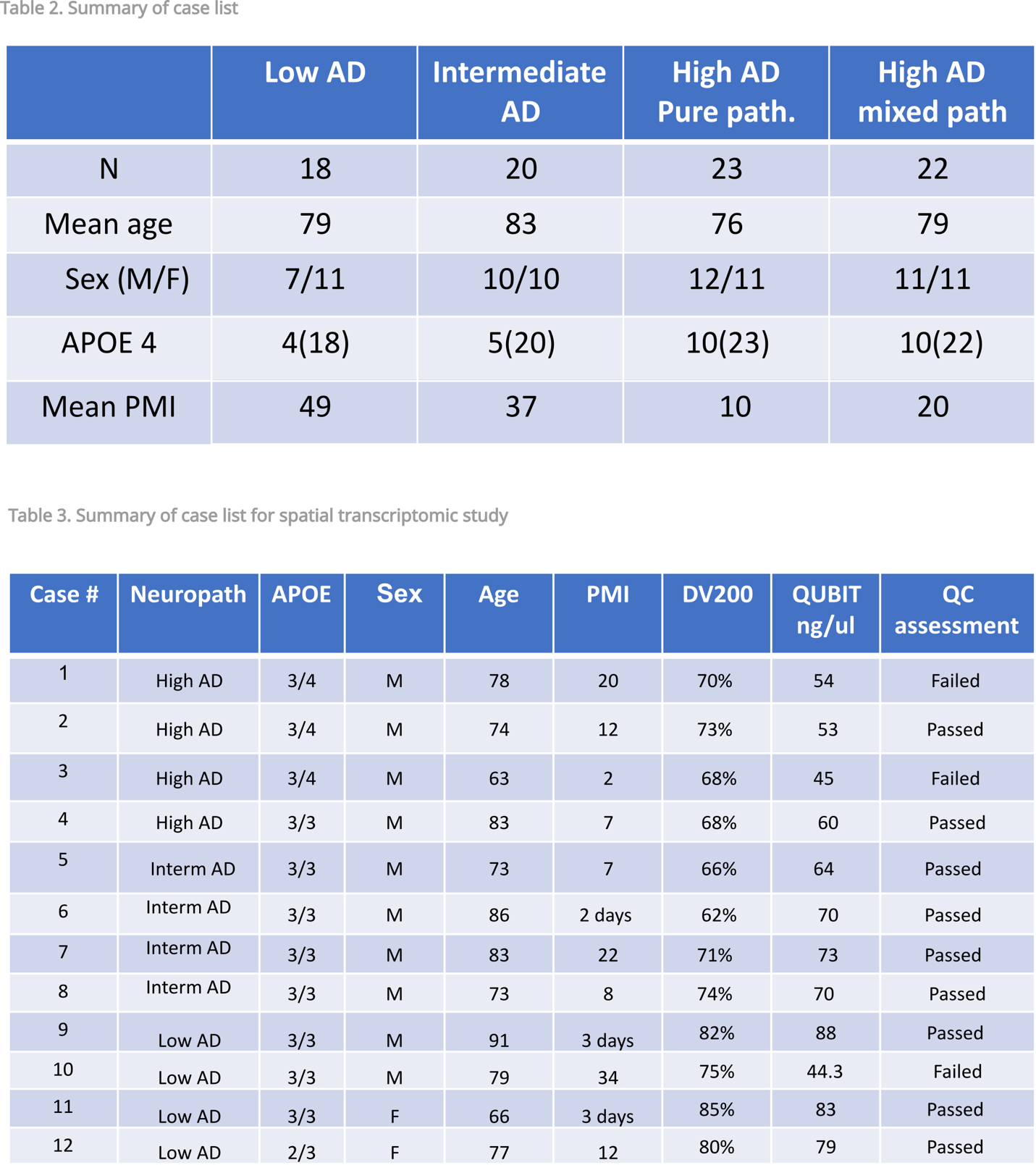


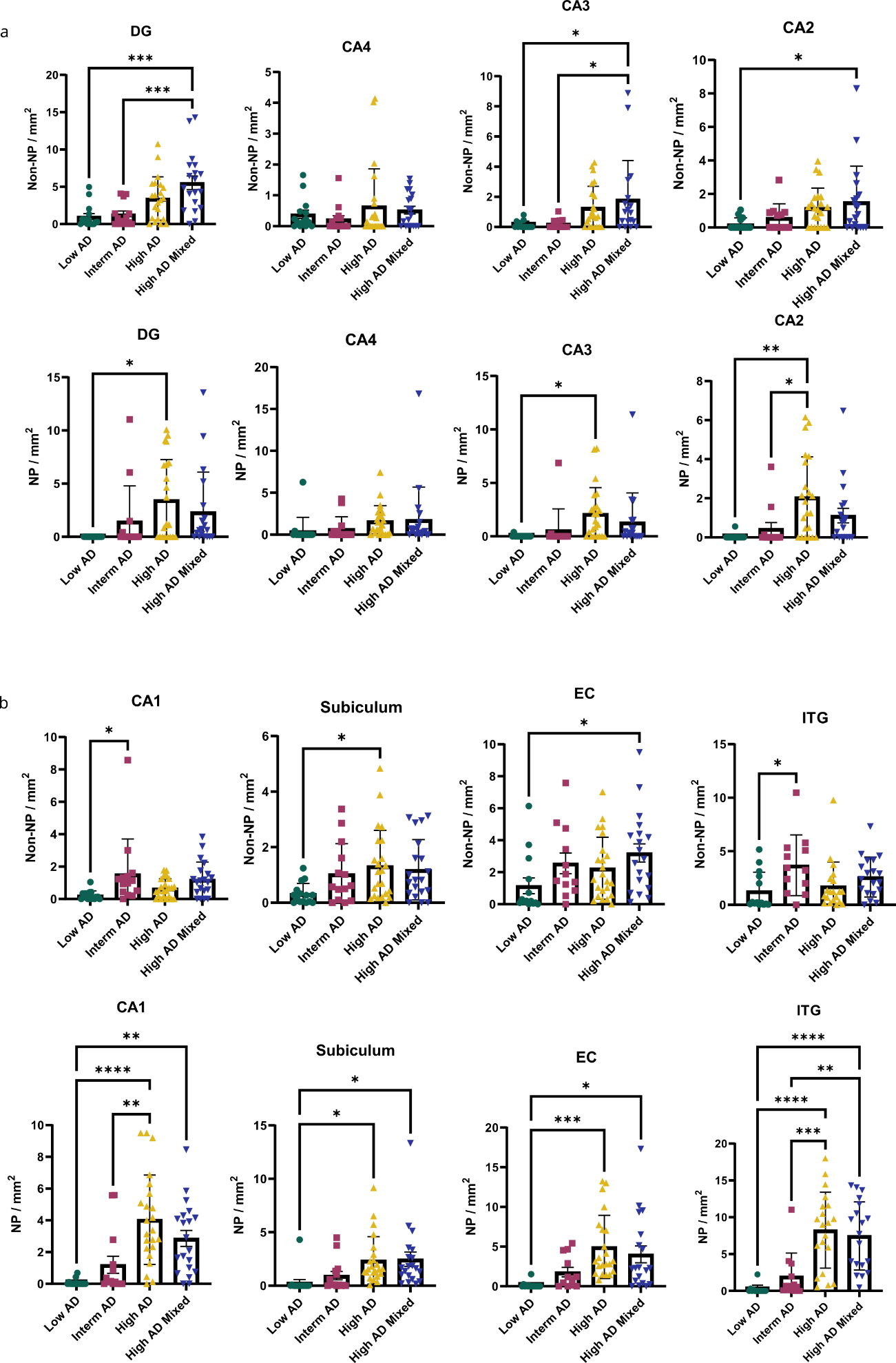


**Supplementary Fig 1**

**Non-NP and NP counts of hippocampal subregions in the progression of ADNC**. (a) NP and non-NP were determined by using modified Gallyas silver staining and anti-Aβ antibody Ab5 (anti-Mouse). Non-NP and NP count was manually counted by blinded observer from the same case and compared between Low AD (n= 15), Interm AD (n= 15), High AD (n=23) and High AD Mixed with co-pathology (n=20).


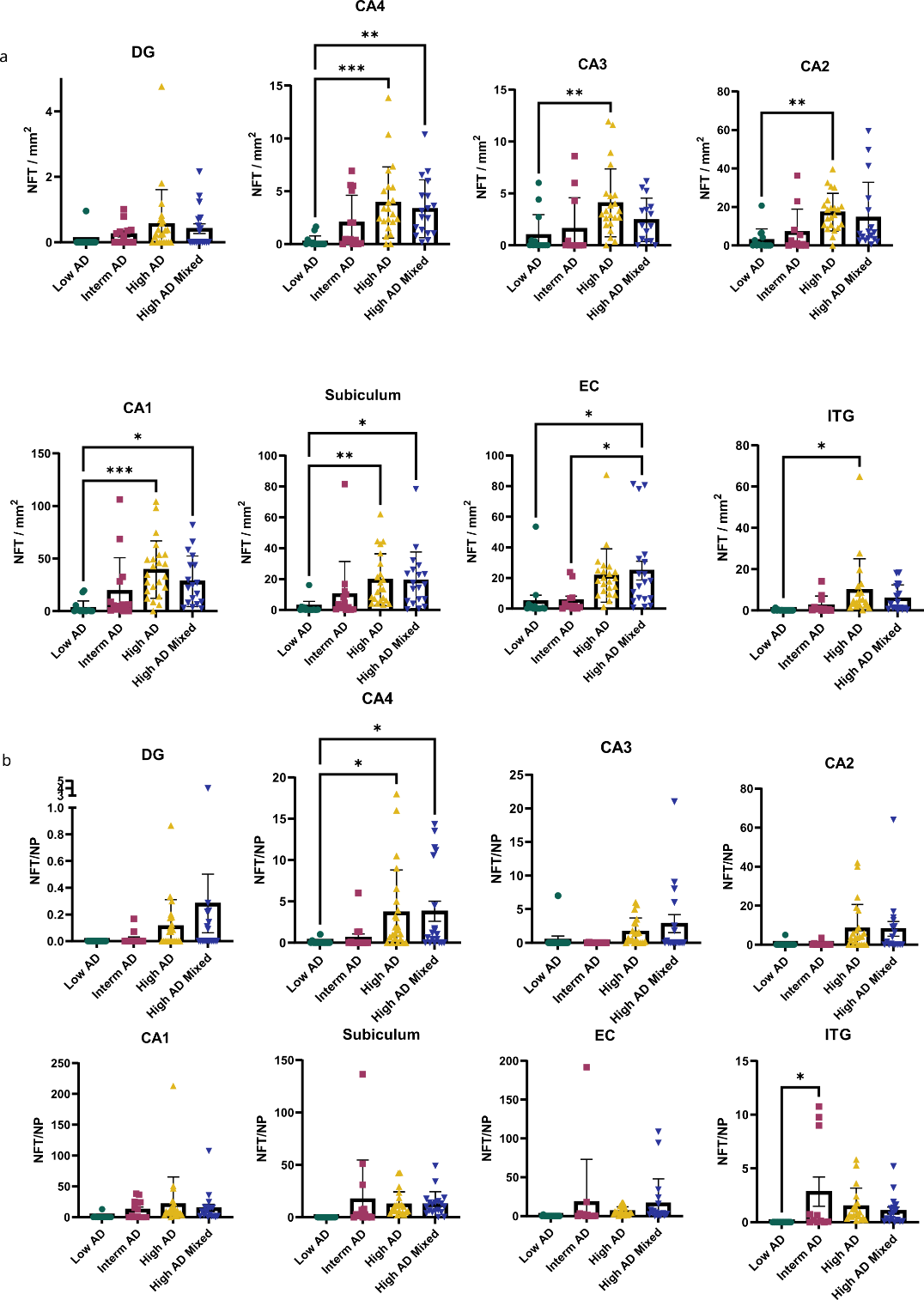


**Supplementary Fig 2.**

**NFT count and NFT/NP ratio of hippocampal subregions in the progression of ADNC.**

Quantification of (a) NFT count (NFT/mm^2^) and (b) NFT/NP ratio in hippocampal subregions. NFT count, along with non-NP and NP count is quantified from the same sample using modified Gallyas silver staining and anti-Aβ antibody Ab5 (anti-Mouse). Sample size - Low AD (n=14), Interm AD (n=15), High AD (n=21) and High AD Mixed with co-pathology (n=20).

**
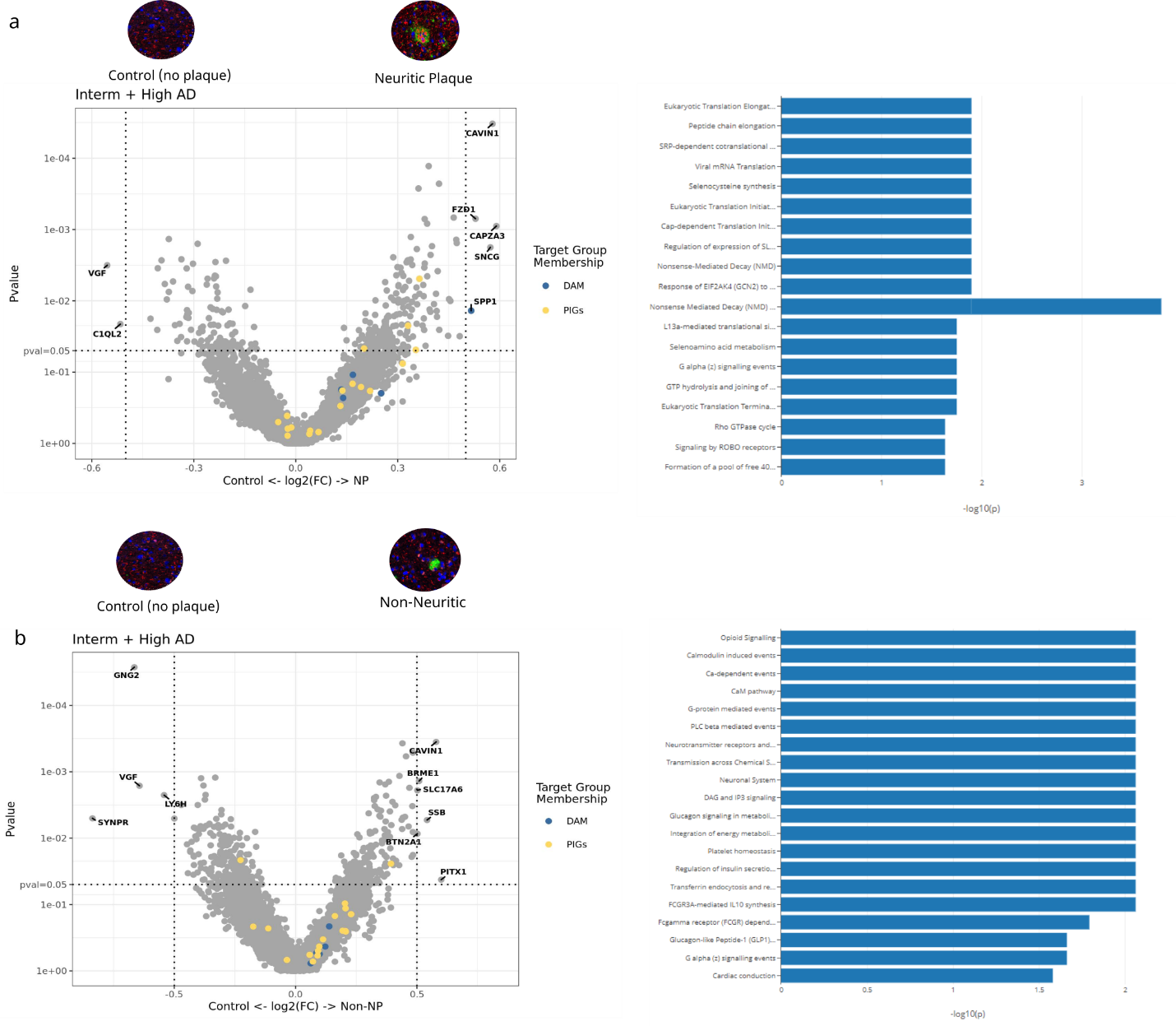
**

**Supplementary Fig 3**.

**Spatial transcriptomic analysis**

1. Volcano plot and pathway analysis comparing control microenvironment and NP microenvironment. Top 20 pathways that are upregulated in both control (microenvironment) and NP microenvironment. The blue color represents pathways that are upregulated in Control (no plaque) or downregulated in NP. The red color represents pathway that are upregulated in NP.
2. Volcano plot and pathway analysis comparing control microenvironment and Non-NP microenvironment. Top 20 pathways that are upregulated in both Control and Non-NP microenvironment. The blue color represents pathways that are upregulated in Control (no plaque) or downregulated in Non-NP. The red color represents pathways that are upregulated in Non-NP.

**
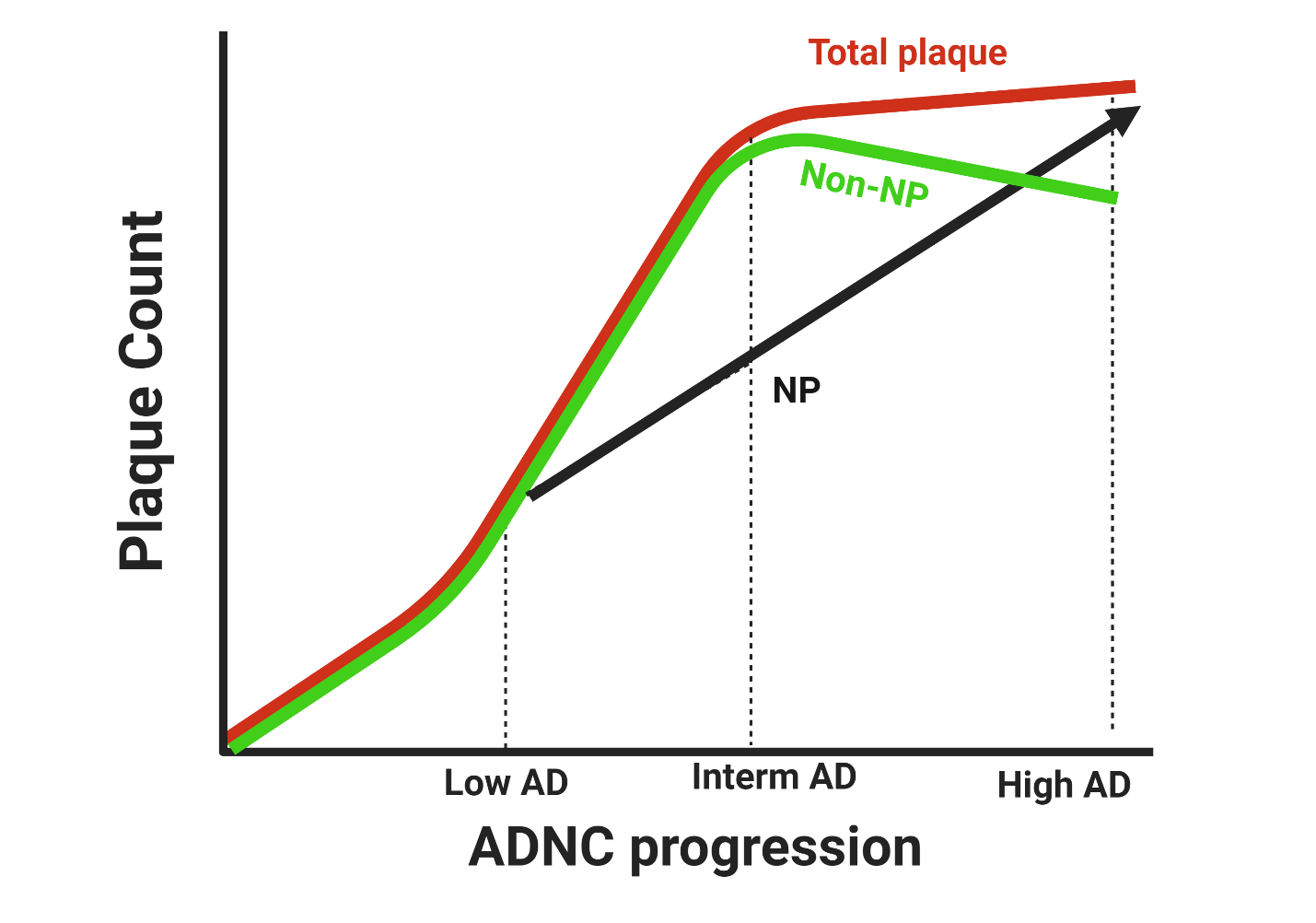
**

**Supplementary Fig 4**

NP increased linearly in ADNC progression (although PHF/tau + NP is rarely observed in Low AD cases), while the non-NP increase from Low to Intermediate stage and then stagnated. Total plaque count increases from Low to Intermediate stage and then plateaus. We hypothesized that non-NP when reaching a certain threshold triggers/transforms into NP, which drives cortical spreading of NFT and cognitive decline.

**
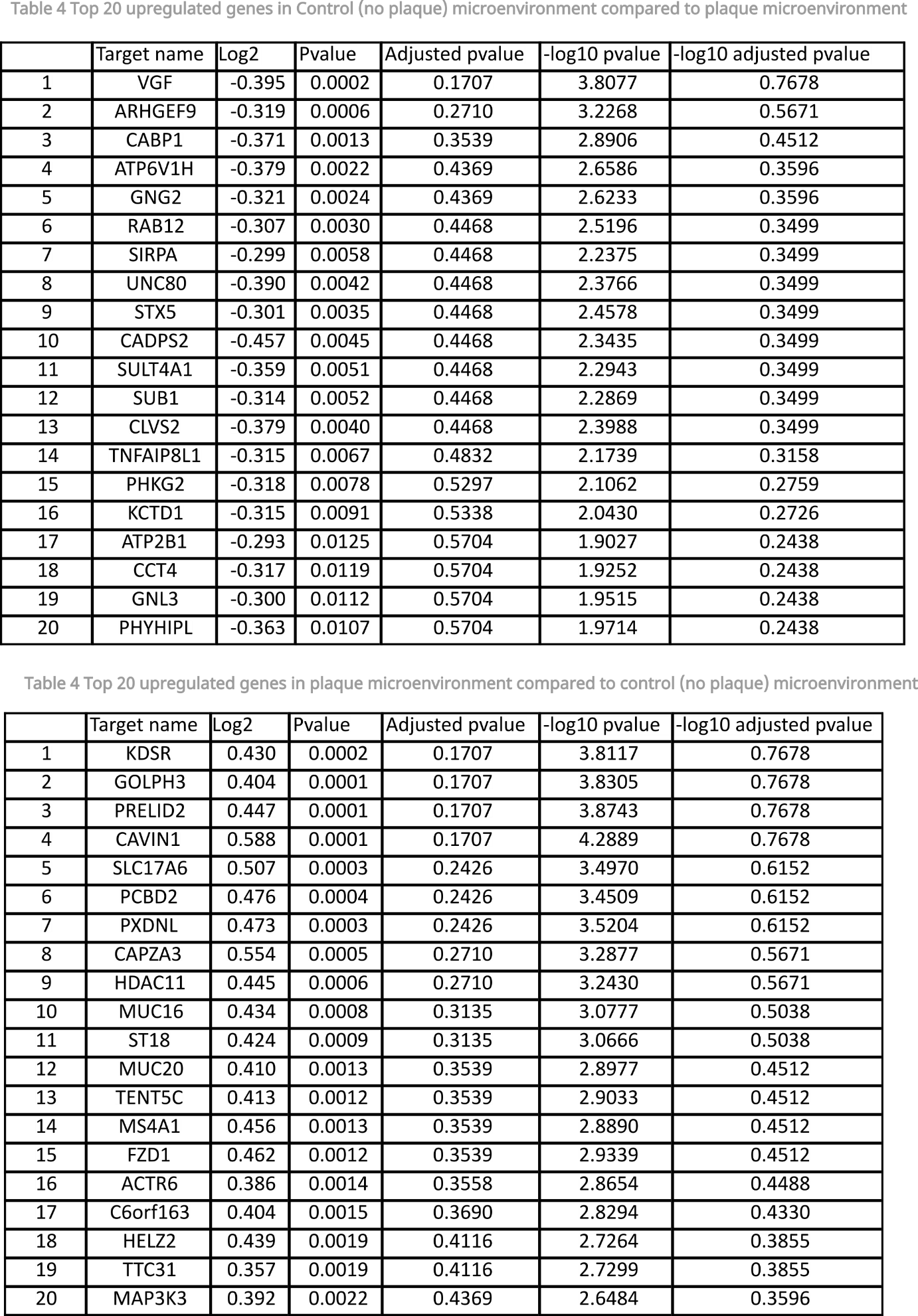
**

**
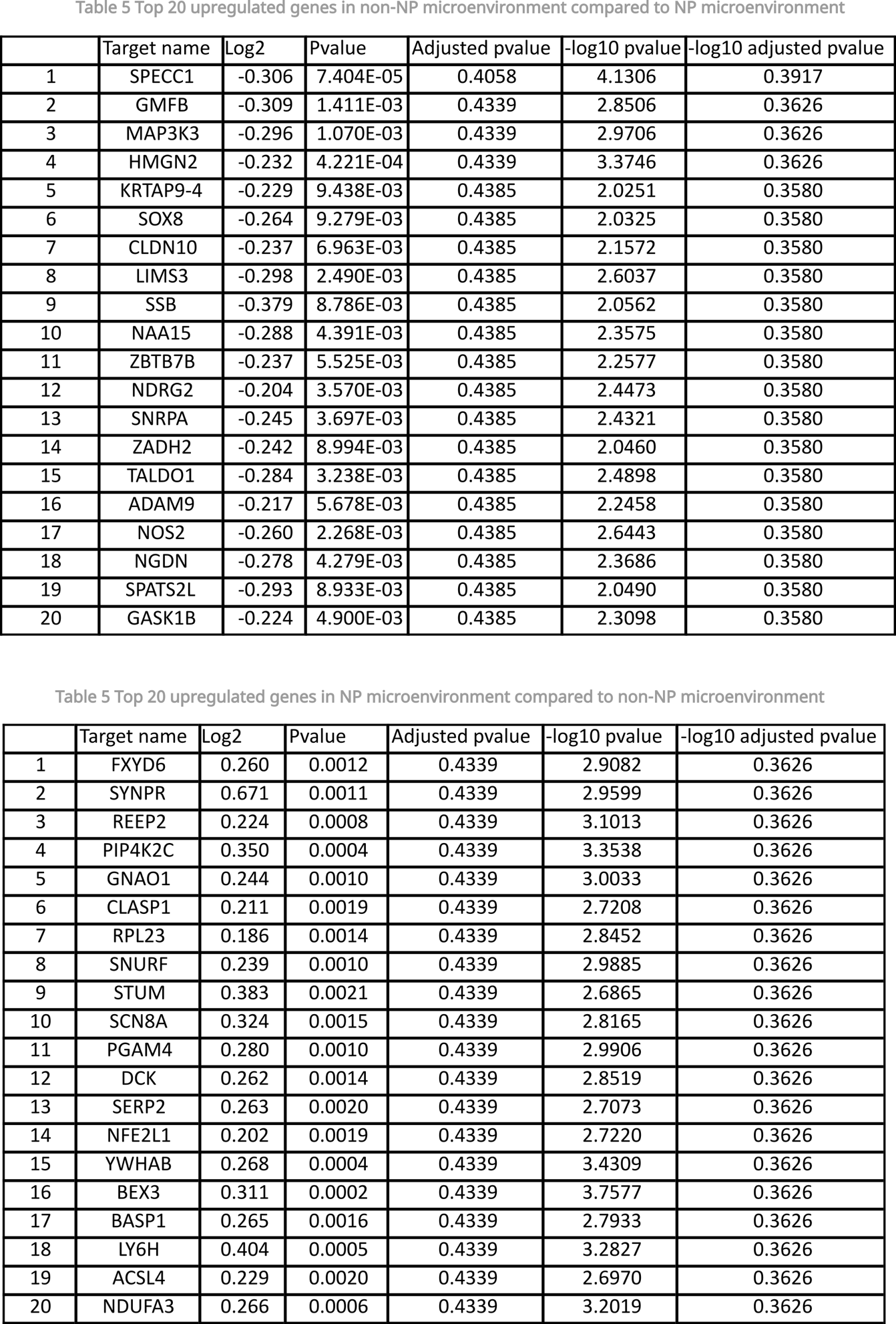
**
